# The impact of changes in COVID‐19 lockdown restrictions on alcohol consumption and drinking occasion characteristics in Scotland and England in 2020: an interrupted time‐series analysis

**DOI:** 10.1111/add.15794

**Published:** 2022-02-02

**Authors:** Iain Hardie, Abigail K. Stevely, Alessandro Sasso, Petra S. Meier, John Holmes

**Affiliations:** ^1^ MRC/CSO Social and Public Health Sciences Unit University of Glasgow Glasgow UK; ^2^ Sheffield Alcohol Research Group (SARG), School of Health and Related Research University of Sheffield Sheffield UK

**Keywords:** Alcohol consumption, COVID‐19 pandemic, drinking occasion characteristics, interrupted time‐series analysis, lockdown restrictions, policy analysis

## Abstract

**Background and Aims:**

Early evidence suggests that COVID‐19 lockdown restrictions affect alcohol consumption. However, existing studies lack data on how drinking practices changed as restrictions disrupted people’s work, family life and socializing routines. We examined changes in consumption and drinking occasion characteristics during three periods of changing restrictions in Scotland/England.

**Design:**

Interrupted time‐series analysis of repeat cross‐sectional market research data (assessing step‐level changes).

**Setting:**

Scotland/England, January 2009–December 2020.

**Participants:**

Scotland: 41 507 adult drinkers; England: 253 148 adult drinkers.

**Measurements:**

Three intervention points: March 2020 lockdown, July 2020 easing of restrictions and October 2020 re‐introduction of some restrictions. Primary outcome: mean units consumed per week (total/off‐trade/on‐trade; 1 unit = 8 g ethanol). Secondary outcomes: drinking > 14 units per week, heavy drinking, drinking days per week, solitary drinking, drinking with family/partners, drinking with friends/colleagues, own‐home drinking, drinking in someone else’s home and drinking start times.

**Findings:**

In Scotland, March 2020’s lockdown was associated with a 2.32 [95% confidence interval (CI) = 0.61, 4.02] increase in off‐trade (i.e. shop‐bought) units per week, a −2.84 (95% CI = −3.63, −2.06) decrease in on‐trade (i.e. licensed venues) units per week, but no statistically significant change in total units per week. July 2020’s easing of restrictions was associated with a 1.33 (95% CI = 0.05, 2.62) increase in on‐trade units per week, but no statistically significant total/off‐trade consumption changes. October 2020’s re‐introduction of some restrictions was not associated with statistically significant consumption changes. Results for England were broadly similar. Lockdown restrictions were also associated with later drinking start times, fewer occasions in someone else’s home and with friends/colleagues, more own‐home drinking and (in Scotland only) more solitary drinking.

**Conclusions:**

Reductions in on‐trade alcohol consumption following COVID‐19 lockdown restrictions in Scotland/England in 2020 were mainly offset by increased own‐home drinking. This largely persisted in periods of greater/lesser restrictions. The shift towards off‐trade drinking involved significant changes in the characteristics of drinking occasions.

## INTRODUCTION

COVID‐19 has led to many countries implementing ‘lockdown’ measures to reduce social contact, e.g. by closing work‐places/businesses/schools and restricting people’s movements and freedom to meet non‐household members face‐to‐face. This has significantly impacted upon health outcomes and health‐related behaviours [1, 2, 3, 4]. With regard to alcohol use, lockdown measures can affect drinking practices in various ways, with hospitality closures and changes to people’s work, family life and socializing routines all likely to alter consumption patterns. Consumption may increase due to stress arising from uncertainty, social isolation and/or loss of work during the pandemic [5] or due to less structured days for those in job retention schemes [6]. Conversely, consumption may decrease due to reduced disposable incomes, less socializing and hospitality closures affecting alcohol availability. Lockdowns may also act as a catalyst for some people to attempt health improvements by re‐evaluating their relationship with alcohol [7].

In the United Kingdom, lockdown restrictions were in place for longer than in many other countries in 2020 due to its comparatively high COVID‐19 case/mortality rates [8]. Restrictions were first introduced in March 2020 and have subsequently changed significantly over time (in response to fluctuating case numbers) and varied between the United Kingdom’s four devolved nations. Key lockdown policy developments in Scotland/England in 2020 are detailed in Table 1. To summarize, the United Kingdom introduced a strict national lockdown in March 2020 to close on‐trade premises (e.g. pubs, bars, clubs and restaurants) and prevent people from leaving their homes except for essential work/shopping or short periods of exercise. Alcohol remained available throughout the year to purchase ‘off‐trade’, i.e. via supermarkets, off‐licences or on‐line deliveries. The UK’s national and devolved governments began significantly easing lockdown restrictions in July 2020 to allow limited indoor gathering and re‐opening of on‐trade premises. However, restrictions (including closures of some on‐trade premises) were then gradually re‐introduced throughout late September–October 2020, with new tiered systems of localized restrictions being introduced, meaning restrictions varied between local areas. England (but not Scotland) then had a second national lockdown in November/December 2020, after which the tiered system returned. In general, lockdown measures tended to be slightly stricter in Scotland than in England, apart from during England’s second lockdown (Table 1).

**TABLE 1:** Key dates/policy developments in the introduction, easing and re‐introduction of COVID‐19 lockdown restrictions in Scotland and England in 2020

Date	UK‐wide lockdown policy developments	Scotland‐specific lockdown policy developments	England‐specific lockdown policy developments
12 March	Those with COVID‐19 symptoms advised to isolate for 7 days		
16 March	Those with COVID‐19 symptoms (and their household) advised to isolate for 14 days Everyone (particularly most vulnerable) advised against all unnecessary social contact. Advice to work from home and avoid on‐trade premises and cinemas. Ban on mass gatherings introduced		
20 March	Schools and businesses such as pubs, bars and restaurants closed		
22 March	Shielding measures introduced to advise 1.5 million people at most risk to stay at home for 12 weeks		
23 March	Strict national lockdown introduced to prevent people from leaving their homes except for essential work, essential shopping or exercise		
13 May			First easing of restrictions as people allowed to exercise as much as they like, drive to outdoor spaces and meet one person from outside their household. Workers from certain sectors encouraged to return to work if their work‐place is open
28 May		Lockdown begins to ease as people can now meet outdoors in groups of up to eight from two households	
1 June			Schools re‐open for some children and people can meet outdoors in groups of up to six
13 June			Single adult households can form a ‘support bubble’
15 June			Non‐essential shops, zoos, safari parks and drive‐in cinemas allowed to re‐open
29 June		Most shops allowed to re‐open, single adult households can form ‘extended households’ and people can meet outdoors with two other households at the same time	
4 July			Two households allowed to meet indoors and pubs/restaurants re‐open (with reduced capacity/physical distancing measures). Tourism and leisure facilities re‐open
6 July		Pubs and cafes are allowed to re‐open (outdoors only and with physical distancing)	
10 July		Indoor gatherings of up to eight people from three households allowed	
15 July		Pubs, restaurants and cafes allowed to re‐open indoors. Tourism resumes	
11 August		Schools start to re‐open fully	
1 September			Most schools re‐open
14 September			The ‘rule of six’ is introduced to prevent social gatherings of more than six people (indoors or outdoors)
23 September		With COVID‐19 cases rising, people are banned from meeting other households indoors. Six people from two households may still meet indoors in cafes, pubs and restaurants	
25 September		10 p.m. curfew imposed on pubs, bars and restaurants	
9 October		On‐trade premises banned from serving alcohol indoors (or at all in some high‐prevalence areas) for 16 days	
14 October			A 3‐tier localized system of restrictions is introduced, meaning that restrictions on visiting other households indoors, and on on‐trade alcohol sales, varied between local areas
2 November		A 5‐tier localized system of restrictions is introduced. This meant that rules regarding on‐trade alcohol sales varied between local authorities, but the ban on visiting other households indoors remained in place for all	
5 November			A second national lockdown is introduced in England for 4 weeks (ending on 2 December 2020)
2 December			Second national lockdown ends and tiered system returns
20 December			A new COVID‐19 variant leads to the introduction of a new tier 4 level (similar to restrictions under the November lockdown), which is imposed on some high‐prevalence areas of England
26 December		The whole of mainland Scotland enters a level 4 lockdown in response to the new variant of COVID‐19	

This table has focused upon policy developments affecting all or large parts of Scotland and England, but there have also been local restrictions introduced for specific areas (e.g. Aberdeen/Leicester) prior to the tier system or the other localized restrictions outlined being introduced. Sources of information are the BBC News Website [9] and The Health Foundation’s COVID‐19 policy tracker [10].

Existing evidence regarding the impact of lockdown restrictions on drinking is mixed. Sales data suggest that despite higher off‐trade sales, overall alcohol sales decreased by 6% in both Scotland and England/Wales during the March–July 2020 lockdown [11]. Descriptive analysis of market research data also suggests that overall consumption fell in England, although did not statistically significantly change in Scotland during the early stages of the pandemic [12], while household shopping panel data suggest that British households did not buy more alcohol than expected for the time of year during the March–July 2020 lockdown [13].

Other UK surveys have tended to suggest that during the initial lockdown up to a third of people were drinking more than before, with a similar proportion drinking less [14], but there has been concern over potential increases in high‐risk/binge drinking [1, 15]. This is backed up by more recent research highlighting a polarization in drinking in England during the pandemic, with some people drinking less than before but heavy drinkers consuming more, and evidence of increased alcohol‐related harm [16]. Internationally, some research suggests that lockdowns are associated with decreased alcohol consumption [17] and some suggests increased hazardous alcohol use [18], but the majority of surveys highlight consumption increasing among some groups but decreasing among others [19, 20, 21]. However, many of these surveys have limitations, such as changing data collection methods during the pandemic and using weak measurements [22]. Existing studies have also been unable to investigate how drinking occasion characteristics may have changed. This may have public health implications, given that risks of alcohol‐related harm varies between drinking contexts [23, 24]. Finally, so far studies have tended to only include data on the early months of the pandemic, so do not provide insight into whether consumption changed further as restrictions were eased and/or re‐introduced. This is important to understand, given ongoing speculation concerning whether people revert to pre‐pandemic drinking once restrictions are relaxed or if there are ‘new norms’ [7, 25].

The current study has the following objectives:

1. To assess the impact of introducing, easing and re‐introducing lockdown restrictions on alcohol consumption in Scotland and England in 2020.
2. To assess the impact of introducing, easing and re‐introducing lockdown restrictions on drinking occasion characteristics (in terms of where people drank, who with, and start times of occasions) in Scotland and England in 2020.

## METHODS

### Research design

The natural experimental conditions of lockdown policy, arising from three periods of changing restrictions over time, were exploited to analyse the impact of introducing, easing and re‐introducing restrictions using interrupted time‐series (ITS) analysis. Scotland and England were analysed separately due to differences in the nature of their restrictions (see Table 1).

### Data

Kantar Alcovision data from January 2009–December 2020 was used. Alcovision is a repeat cross‐sectional on‐line survey with an annual sample of approximately 30 000 adults in Great Britain. It draws weekly quota samples from an on‐line market research panel, with quotas based on gender, social class, age and geographic region to match nationally representative targets. Samples are drawn continuously throughout the year, with Scotland being oversampled to permit robust analysis. To increase representativeness, we used a ‘raking’ weighting technique which calibrates survey weights to UK census data (see Supporting information, Appendix A for full details).

Alcovision comprises a short introductory questionnaire and a detailed retrospective 7‐day drinking diary, which gathers information on respondents’ alcohol consumption and drinking occasion characteristics. Drinking occasions are defined as a significant time‐period (e.g. lunchtime, early evening or late evening). Respondents can report up to two on‐trade occasions and two off‐trade occasions per day.

The final analytical sample throughout the study period was 41 507 individuals in Scotland and 253 148 individuals in England. Respondents in Wales were excluded due to their small sample size (14 556 throughout the study period). Respondents who abstain from alcohol, i.e. report drinking less than once per year (5773 individuals in Scotland and 36 266 individuals in England throughout the study period) were also excluded from the analysis.

### Measures

#### Intervention points

Key dates in lockdown policy developments were used to specify three intervention points: (1) initial lockdown, i.e. March 2020, when lockdown measures were first introduced, (2) restrictions easing, i.e. July 2020, when on‐trade premises reopened and indoor household mixing rules were relaxed, and (3) some restrictions re‐introduced, i.e. October, 2020 when local restrictions and the localized tiered systems were introduced (and also covering the period of England’s second lockdown). Each intervention point was coded as 0 before the month of the intervention point and 1 during/after the month of the intervention point. Intervention points were the same for Scotland and England as, although restrictions varied between countries, timings of policy developments were broadly similar (see Table 1).

#### Outcome measures

To provide an overall picture of consumption the primary outcome was mean units per week, which was split into total/off‐trade/on‐trade to offer insight into substitution between settings. This is the mean number of units that respondents reported consuming in their 7‐day drinking diary. Alcovision records consumption in ‘serves’. These were converted into UK units (1 unit = 8 g ethanol) using information on packaging size, drink type and alcohol by volume (ABV). To prevent unrealistically high values biasing the results, individual reports are capped at 280 units per week. Full details of these processes are provided in Supporting information, Appendix A.

Secondary outcomes include three further consumption measures and six measures of drinking occasion characteristics. Consumption measures were: (1) proportion of individuals drinking > 14 units per week, i.e. the proportion consuming more than recommended by UK drinking guidelines during the diary week, (2) mean number of heavy drinking occasions per week, i.e. the mean number of occasions per diary week involving > 6 units for women or > 8 units for men and (3) mean number of drinking days per week, i.e. the mean number of diary week days in which respondents reported drinking. These consumption measures complement the primary outcome by providing insight into whether people were drinking more often/more heavily during the pandemic, over which previous studies have raised concerns [1, 18]. Like the primary outcome, these were all split into total/off‐trade/on‐trade. Occasion characteristics measures include three measures of who occasions were with, two measures of off‐trade locations and one measure of occasion start times. They are: (1) mean number of solitary occasions per week, (2) mean number of occasions per week with family/partner, (3) mean number of occasions per week with friends/colleagues, (4) mean number of occasions per week in own home, (5) mean number of occasions per week in someone else’s home and (6) mean start time of first drinking occasion per day. All measures were selected following the results of previous descriptive analysis [12].

### Statistical analysis

First, descriptive analysis was conducted to assess how alcohol consumption and drinking occasion characteristics varied throughout 2020. This was performed by plotting each outcome over time in 2020 and, for comparison with recent years, comparing with the 2016–19 average for the same month.

The impact of changes in lockdown restrictions was then more formally evaluated via ITS analysis, using data from the full January 2009–December 2020 time‐series. July 2017 was missing, which was handled using Kalman filtering [26]. The analytical process involved constructing a monthly time‐series, whereby individual‐level Alcovision data were aggregated to give monthly averages for each outcome. Seasonal autoregressive moving average (SARMA) modelling was used to estimate the effect of each change in lockdown restrictions on each outcome, adjusting for autocorrelation, seasonality and trend. Candidate SARMA models were selected by testing for autocorrelation and non‐stationarity using auto‐correlation function (ACF) and partial auto‐correlation function (PACF). The most appropriate models were then selected by using Akaike information criteria (AIC) and Bayesian information criteria (BIC), and testing model assumptions. All analyses were undertaken on weighted data and conducted using Stata/MP version 16.1. This analytical protocol was not pre‐registered. As such, results should be considered exploratory.

## RESULTS

Table 2 gives descriptive statistics on the mean values of each outcome pre‐lockdown and during each period of changes in restrictions. It also provides information on the number of individuals in the sample in each country‐period.

**TABLE 2:** Sample size and weighted mean of outcome measures in each country‐period

		Pre‐lockdown		Initial lockdown		Restrictions eased		Some restrictions re‐introduced	
		Scotland	England	Scotland	England	Scotland	England	Scotland	England
*n*		38 682	236 405	1169	6789	824	4993	832	4961
Alcohol consumption									
Total	Mean units per week	17.0	16.5	14.3	13.7	15.3	15.0	13.8	14.5
	Proportion of individuals drinking > 14 units per week (%)	38.7%	37.5%	31.6%	30.5%	32.5%	33.3%	29.4%	34.2%
	Mean number of heavy drinking occasions per week	0.9	0.8	0.7	0.6	0.7	0.7	0.7	0.7
	Mean number of drinking days per week	1.7	2.0	1.5	1.6	1.6	1.7	1.5	1.7
Off‐trade	Mean units per week	11.9	11.4	12.6	11.8	12.4	11.9	11.9	12.0
	Proportion of individuals drinking > 14 units per week (%)	27.8%	26.7%	27.5%	26.7%	26.6%	26.7%	26.0%	28.7%
	Mean number of heavy drinking occasions per week	0.6	0.5	0.6	0.6	0.6	0.6	0.6	0.6
	Mean number of drinking days per week	1.5	1.7	1.4	1.5	1.4	1.5	1.4	1.5
On‐trade	Mean units per week	5.2	5.1	1.7	1.9	2.9	3.2	1.9	2.5
	Proportion of individuals drinking > 14 units per week (%)	12.4%	11.9%	4.7%	4.6%	6.3%	7.2%	3.8%	6.1%
	Mean number of heavy drinking occasions per week	0.3	0.2	0.8	0.9	0.1	0.1	0.1	0.1
	Mean number of drinking days per week	0.5	0.6	0.1	0.2	0.3	0.4	0.2	0.2
Drinking occasion characteristics									
Who with	Mean number of solitary occasions per week	0.4	0.5	0.5	0.5	0.4	0.5	0.5	0.5
	mean number of occasions per week with	1.3	1.6	1.0	1.3	1.2	1.3	1.0	1.2
	family/partner mean number of occasions per week with friends/colleagues	0.8	0.8	0.3	0.3	0.5	0.5	0.3	0.4
Off‐trade location	Mean number of occasions per week in own home	1.3	1.6	1.5	1.6	1.4	1.5	1.4	1.5
	Mean number of occasions per week in someone else’s home	0.3	0.3	0.1	0.1	0.2	0.2	0.1	0.2
Start time	Mean start time of first drinking occasion per day	18:05	17:53	18:25	18:04	18:10	17:46	18:16	18:07

Pre‐lockdown covers the period January 2009–February 2020, initial lockdown covers the period March 2020–June 2020, restrictions eased covers the period July 2020–September 2020 and some restrictions re‐introduced covers the period some off‐trade occasions took place in locations other than respondent’s own home or someone else’s home (e.g. outdoors, in holiday accommodation, outdoors or an event). However, these were not included in the analysis due to making up a very small proportion of off‐trade occasions overall.

**FIGURE 1:**
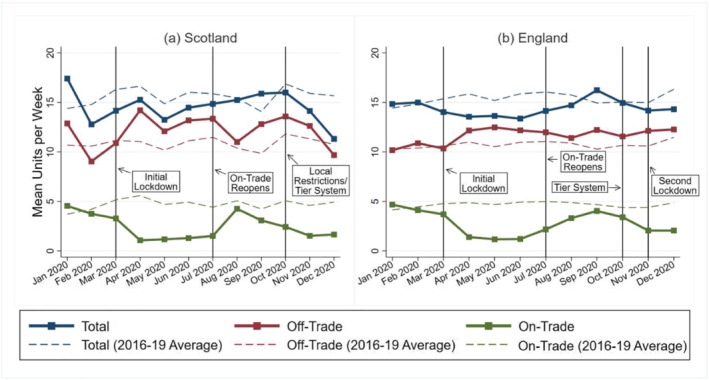
Mean units per week in Scotland and England in 2020 (with 2016–19 average for comparison). Note: The re‐opening of on‐trade premises also coincided with the relaxation of restrictions around mixing with other households indoors

**FIGURE 2:**
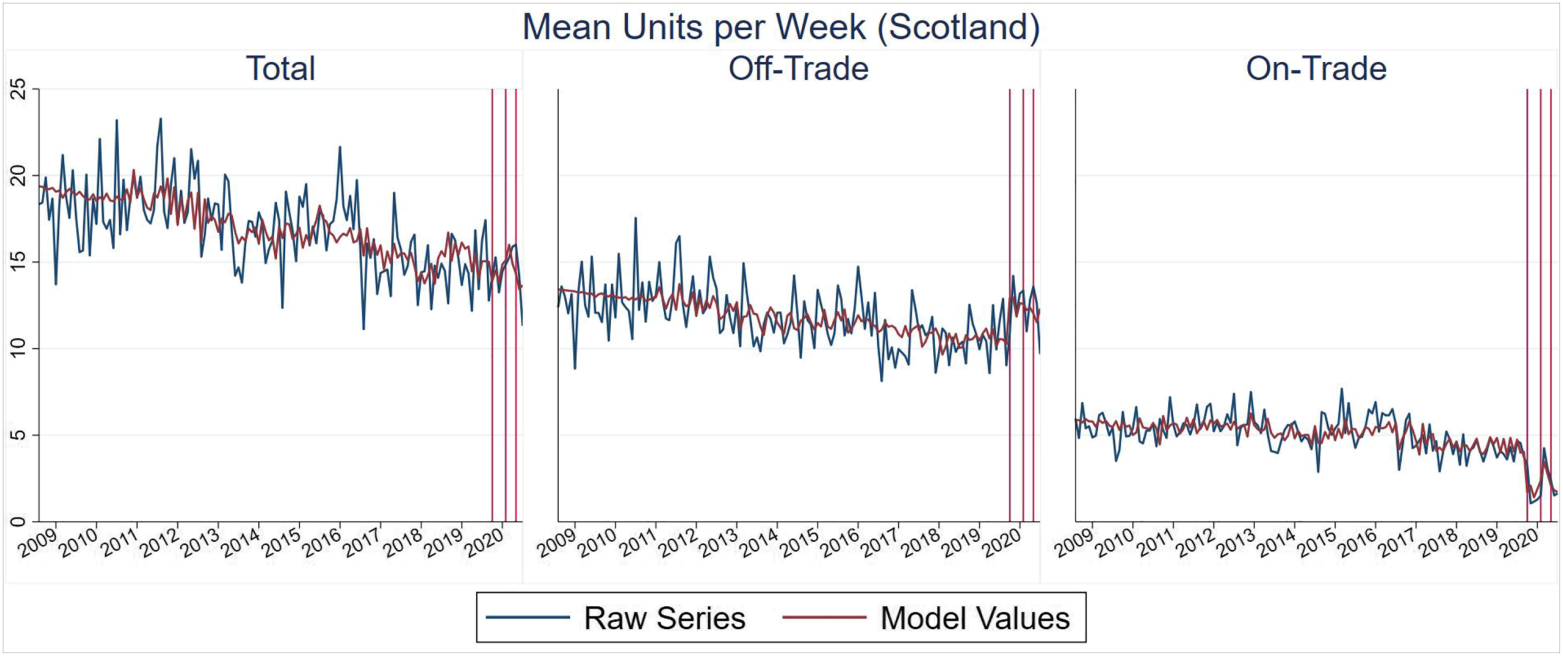
Monthly mean units per week in Scotland (vertical lines from left to right = months where lockdown restrictions were introduced, eased and re‐introduced)

**FIGURE 3:**
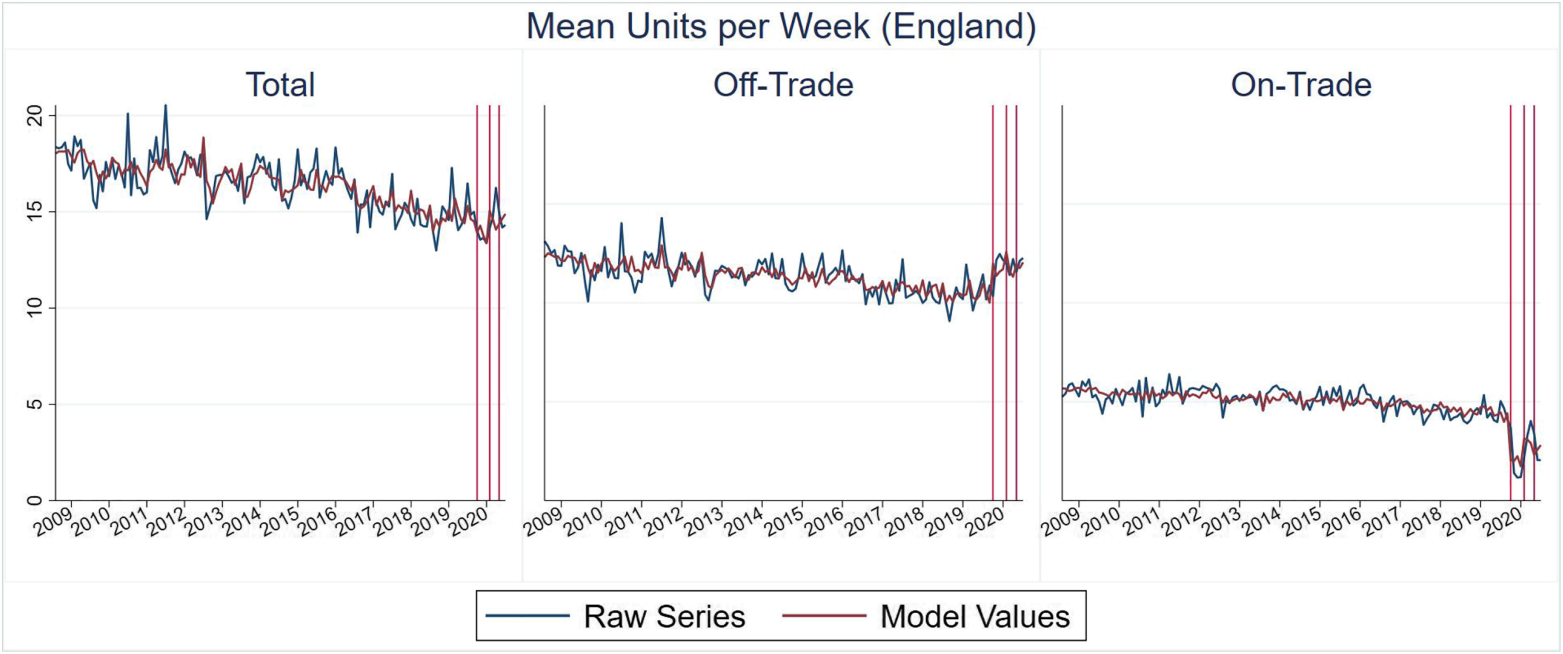
Monthly mean units per week in England (vertical lines from left to right = months where lockdown restrictions were introduced, eased and re‐introduced)

The descriptive analysis results are provided in Figure 1 (for the primary outcome) and Supporting information, Appendix B (for secondary outcomes). The ITS analysis results are provided in Tables 3, 4, 5, 6. Model values are shown visually alongside the raw monthly time‐series in Figures 2 and 3 (for the primary outcome) and Supporting information, Appendix C (for secondary outcomes). Supporting information, Appendix C also provides truncated versions of all model values/raw series figures, which allow for easier interpretation of the COVID‐19 period.

**TABLE 3:** Estimated impact of the introducing, easing and re‐introducing COVID‐19 lockdown restrictions on alcohol consumption measures in Scotland (step changes)

	(1) Mean units per week		(2) Proportion of individuals drinking >14 units per week		(3) Mean number of heavy drinking occasions per week		(4) Mean number of drinking days per week	
Total	B (95% CI)	*P*	B (95% CI)	*P*	B (95% CI)	*P*	B (95% CI)	*P*
Initial lockdown	−0.84	0.781	−0.02	0.573	−0.05	0.569	0.07	0.594
	(−6.76, 5.09)		(−0.09, 0.05)		(−0.20, 0.11)		(−0.18, 0.31)	
Restrictions eased	1.18	0.765	0.01	0.849	0.05	0.870	0.10	0.270
	(−6.54, 8.89)		(−0.08, 0.10)		(−0.52, 0.61)		(−0.44, 0.64)	
Some restrictions re‐introduced	−1.64	0.557	−0.04	0.351	−0.05	0.857	−0.08	0.758
	(−7.11, 3.83)		(−0.12, 0.04)		(−0.61, 0.50)		(−0.61, 0.45)	
Constant	39.63	< 0.001	0.84	< 0.001	1.72	< 0.001	4.63	< 0.001
	(35.25, 44.01)		(0.72, 0.96)		(1.44, 2.00)		(4.26, 5.00)	
AR terms	L23: −0.26	0.003	L4: 0.18	0.023	L30: −0.25	0.013	L18: −0.17	0.047
	(−0.43, −0.09)		(0.02, 0.33)		(−0.45, −0.05)		(−0.40, −0.002)	
	L25: −0.26	0.002	L30: −0.23	0.046				
	(−0.43, −0.09)		(−0.46, −0.003)					
Off‐trade								
Initial lockdown	2.32	0.008	0.03	0.118	0.09	0.017	0.23	0.002
	(0.61, 4.02)		(−0.01, 0.07)		(0.02, 0.16)		(0.08, 0.37)	
Restrictions eased	−0.08	0.962	−0.00	0.886	0.01	0.819	0.02	0.912
	(−3.13, 2.98)		(−0.07, 0.06)		(−0.10, 0.12)		(−0.28, 0.32)	
Some restrictions re‐introduced	−0.61	0.679	−0.01	0.807	−0.02	0.770	−0.01	0.942
	(−3.48, 2.26)		(−0.08, 0.06)		(−0.15, 0.10)		(−0.35, 0.32)	
Constant	27.21	< 0.001	0.63	< 0.001	1.08	< 0.001	3.94	< 0.001
	(23.58, 30.83)		(0.54, 0.72)		(0.86, 1.36)		(3.61, 4.28)	
AR terms	L25: −0.18	0.044	L25: −0.19	0.082	L30: −0.20	0.039	L18: −0.18	0.047
	(−0.36, −0.01)		(−0.30, 0.02)				(−0.35, −0.002)	
	L31: −0.21	0.034						
	(−0.40, −0.02)							
On‐trade								
Initial lockdown	−2.84	< 0.001	−0.06	< 0.001	−0.14	< 0.001	−0.29	< 0.001
	(−3.63, −2.06)		(−0.09, −0.04)		(−0.20, −0.09)		(−0.35, −0.23)	
Restrictions eased	1.33	0.041	0.02	0.208		0.034	0.14	0.008
	(0.05, 2.62)		(−0.01, 0.06)		0.07		(0.04, 0.25)	
Some restrictions re‐introduced	−0.88	0.601	−0.03	0.437	(0.01, 0.13)	0.454	−0.10	0.202
	(−4.16, 2.41)		(−0.09, 0.04)		−0.05		(−0.26, 0.05)	
Constant	12.23	< 0.001	0.30	< 0.001	(−0.19, 0.08)	< 0.001	1.40	< 0.001
	(7.81, 16.63)		(0.22, 0.38)		0.64		(0.99, 1.80)	
AR terms	L1: 0.18	0.041	L2: 0.16	0.081	(0.45, 0.84)	0.036	L1: 0.17	0.046
	(0.01, 0.35)		(−0.02, 0.34)		L11: 0.19		(0.003, 0.34)	
	L5: 0.18	0.046	L23: −0.25	0.004	(0.01, 0.37)	0.011	L11: 0.19	0.067
	(0.002, 0.36)		(−0.42, −0.08)		L20: −0.21		(−0.01, 0.39)	
	L23: −0.29	0.001			(−0.37, −0.48)			
	(−0.46, −0.13)							
Seasonal (12) AR terms	L1: 0.16	0.086	L1: 0.20	0.036		0.013	L1: 0.25	0.006
	(−0.02, 0.34)		(0.01, 0.38)				(0.07, 0.44)	
	L2: 0.26	0.007			L1: 0.24			
	(0.07, 0.45)				(0.05, 0.42)			

Estimates are adjusted for autocorrelation, seasonality and trend. B = coefficient, 95% confidence intervals (CI) and *P* = *P*‐value. ‘Initial lockdown’ includes all diary weeks from March 2020 onwards, ‘Restrictions eased’ includes all diary weeks from July 2020 (when on‐trade premises reopened and restrictions on visiting other households were relaxed) onwards and ‘Some restrictions re‐introduced’ includes all diary weeks from October 2020 (when the tier system was introduced, and also covering the period of England’s second lockdown from November 2020) onwards. Results for the AR terms and seasonal AR terms included in each model are reported underneath the main results. L refers to number of lags, and these were selected following an iterative process involving autocorrelation function/autocorrelation function (ACF/PACF) plots and model fit statistics.

**TABLE 4:** Estimated impact of the introducing, easing and re‐introducing COVID‐19 lockdown restrictions on alcohol consumption measures in England (step changes)

	(1) Mean units per week		(2) Proportion of individuals drinking > 14 units per week		(3) Mean number of heavy drinking occasions per week		(4) Mean drinking days per week	
Total	B (95% CI)	*P*	B (95% CI)	*P*	B (95% CI)	*P*	B (95% CI)	*P*
Initial lockdown	−0.95	0.434	−0.02	0.404	−0.05	0.486	−0.03	0.715
	(−3.34, 1.43)		(−0.06, 0.02)		(−0.20, 0.09)		(−0.16, 0.11)	
Restrictions eased	1.07	0.325	0.03	0.132	0.08	0.297	0.05	0.573
	(−1.06, 3.21)		(−0.01, 0.07)		(−0.07, 0.23)		(−0.13, 0.23)	
Some restrictions re‐introduced	−0.56	0.611	0.01	0.976	−0.05	0.305	0.02	0.809
	(−2.27, 1.61)		(−0.38, 0.39)		(−0.15, 0.05)		(−0.14, 0.18)	
Constant	31.90	< 0.001	0.81	< 0.001	1.37	< 0.001	5.01	< 0.001
	(23.50, 40.30		(0.65, 0.96)		(1.12, 1.62)		(3.93, 6.08)	
AR terms	L1: 0.32	< 0.001	L1: 0.19	0.028	L1: 0.41	< 0.001	L1: 0.18	0.062
	(0.16, 0.48)		(0.02, 0.36)		(0.26, 0.56)		(−0.01, 0.36)	
	L2: 0.19	0.043	L2: 0.28	0.001	L13: −0.22	0.001	L2: 0.25	0.011
	(0.01, 0.38)		(0.12, 0.45)		(−0.35, −0.09)		(0.06, 0.44)	
							L3: 0.20	0.024
							(0.03, 0.38)	
Seasonal (12) AR terms	L1: 0.24	0.001	L1: 0.20	0.012		0.001	L1: 0.27	0.006
	(0.10, 0.39)		(0.04, 0.36)		L1: 0.26		(0.08, 0.47)	
	L2: 0.28 (0.09, 0.47)	0.004	L2: 0.25 (0.07, 0.44)	0.007	(0.26, 0.56) L2: 0.24	0.011	L2: 0.06 (−0.17, 0.29)	0.601
					(0.06, 0.43)			
								0.024
							L3: 0.25	
							(0.03, 0.38)	
Off‐trade								
Initial lockdown	1.18	< 0.001	0.03	< 0.001	0.06	0.005	0.11	0.002
	(0.65, 1.70)		(0.02, 0.05)		(0.02, 0.10)		(0.04, 0.18)	
Restrictions eased	0.38	0.445	0.01	0.684	0.03	0.541	0.02	0.953
	(−0.59, 1.34)		(−0.03, 0.05)		(−0.08, 0.15)		(−0.52, 0.56)	
Some restrictions re‐introduced	−0.13	0.877	0.02	0.394	−0.03	0.651	0.01	0.960
	(−1.79, 1.53)		(−0.02, 0.06)		(−0.18, 0.11)		(−0.47, 0.49)	
Constant	20.50	< 0.001	0.61	< 0.001	0.81	< 0.001	4.05	< 0.001
	(17.58, 23.42)		(0.55, 0.67)		(0.67, 0.95)		(3.70, 4.39)	
AR terms	L1: 0.32	< 0.001	L1: 0.20	0.033	L1: 0.29	< 0.001	L1: 0.20	0.017
	(0.16, 0.48)		(0.02, 0.39)		(0.14, 0.44)		(0.04, 0.36)	
	L13: −0.203	0.009			L13: −0.25	< 0.001	L39: −0.39	< 0.001
	(−0.35, −0.05)				(−0.38, −0.12)		(−0.58, −0.21)	
Seasonal (12) AR terms	L1: 0.31	< 0.001			L1: 0.30	< 0.001	L1: 0.34	< 0.001
	(0.14, 0.47)				(0.14, 0.46)		(0.16, 0.52)	
	L2: 0.11	0.360						
	(−0.13, 0.35)							
On‐trade								
Initial lockdown	−2.53	< 0.001	−0.06	< 0.001	−0.12	< 0.001	−0.32	< 0.001
	(−2.86, −2.20)		(−0.06, −0.05)		(−0.14, −0.11)		(−0.32, −0.02)	
Restrictions eased	1.37	< 0.001	0.03	< 0.001	0.06	< 0.001	0.17	< 0.001
	(0.82, 1.91)		(0.01, 0.04)		(0.04, 0.09)		(0.10, 0.24)	
Some restrictions re‐introduced	−0.73	0.002	−0.01	0.068	−0.03	0.019	−0.12	< 0.001
	(−1.19, −0.26)		(−0.03, 0.00)		(−0.06, 0.01)		(−0.18, −0.07)	
Constant	11.19	< 0.001	0.27	< 0.001	0.54	< 0.001	1.50	< 0.001
	(8.63, 13.75)		(0.20, 0.33)		(0.44, 0.64)		(0.97, 2.03)	
AR terms	L2: 0.27	0.006	L2: 0.23	0.008	L2: 0.20	0.034	L1: 0.24	0.001
	(0.08, 0.45)		(0.06, 0.41)		(0.02, 0.39)		(0.10, 0.38)	
							L2: 0.34	< 0.001
							(0.17, 0.50)	
Seasonal (12) AR terms	L1: 0.11	0.294					L1: 0.09	0.449
	(−0.10, 0.32)						(−0.15, 0.34)	
	L2: 0.09	0.513	L1: 0.15	−0.168			L2: 0.28	0.052
	(−0.17, 0.34)		(−0.06, 0.37)				(−0.002, 0.56)	
	L3: 0.27	0.022	L2: 0.11	0.425			L3: 0.23	0.079
	(0.04, 0.50)		(−0.16, 0.37)				(−0.03, 0.49)	
			L3: 0.23	0.085				
			(−0.03, 0.48)					

Estimates are adjusted for autocorrelation, seasonality and trend. B = coefficient, 95% confidence intervals (CI) and *P* = *P*‐value. ‘Initial lockdown’ includes all diary weeks from March 2020 onwards, ‘Restrictions eased’ includes all diary weeks from July 2020 (when on‐trade premises re‐opened and restrictions on visiting other households were relaxed) onwards and ‘Some restrictions re‐introduced’ includes all diary weeks from October 2020 (when the tier system was introduced, and also covering the period of England’s second lockdown from November 2020) onwards. Results for the AR terms and seasonal AR terms included in each model are reported underneath the main results. L refers to number of lags, and these were selected following an iterative process involving autocorrelation function/autocorrelation function (ACF/PACF) plots and model fit statistics.

**TABLE 5:** Estimated Impact of the introducing, easing and re‐introducing COVID‐19 lockdown restrictions on drinking occasion characteristics (who with, off‐trade location and start time) in Scotland (step changes)

	Mean number of occasions per week (by who with)						Mean number of occasions per week (by off‐trade location)				Mean start time of first drinking occasion	
	(1) Solitary		(2) With family/partner		(3) With friends/colleagues		(4) Own home		(5) Someone else’s home		(6) Start time	
	B (95% CI)	*P*	B (95% CI)	*P*	B (95% CI)	*P*	B (95% CI)	*P*	B (95% CI)	*P*	B (95% CI)	*P*
Initial lockdown	0.08	0.005	0.08	0.552	−0.32	< 0.001	0.38	< 0.001	−0.08	0.010	0.59	0.007
	(0.02, 0.14)		(−0.17, 0.32)		(−0.45, −0.20)		(0.25, 0.51)		(−0.14, −0.02)		(0.16, 1.02)	
Restrictions eased	−0.10	0.590	0.13	0.686	0.16	0.102	−0.09	0.568	0.10	0.524	−0.26	0.593
	(−0.48, 0.27)		(−0.51, 0.78)		(−0.03, 0.34)		(−0.42, 0.23)		(−0.21, 0.41)		(−1.22, 0.70)	
Some restrictions re‐introduced	0.11	0.576	−0.14	0.842	−0.17	0.166	0.05	0.808	−0.08	0.681	−0.04	0.924
	(−0.27, 0.27)		(−1.56, 1.27)		(−0.40, 0.07)		(−0.34, 0.43)		(−0.45, 0.30)		(−0.95, 0.86)	
Constant	0.32	0.043	4.47	< 0.001	2.16	< 0.001	3.74		1.04	< 0.001	19.70	< 0.001
	(0.01, 0.63)		(4.00, 4.94)		(1.81, 2.51)		(< 0.001)		(0.81, 1.26)		(19.06, 20.34)	
AR terms	L10: 0.21	0.025	L27: 0.08	0.451	L5: 0.16	0.044	L14: −0.17	0.103	L4: 0.20	0.010	L27: 0.28	0.001
	(0.03, 0.39)		(−0.12, 0.29)		(0.005, 0.33)		(−0.37, 0.03)		(0.05, 0.36)		(0.12, 0.43)	
					L30: −0.29	0.003			L5: 0.21	0.020	L35: −0.26	0.006
					(−0.48, −0.10)				(0.03, 0.38)		(−0.43, −0.07)	

Estimates are adjusted for autocorrelation, seasonality and trend. B = coefficient, 95% confidence intervals (CI) and *P* = *p*‐value. ‘Initial lockdown’ includes all diary weeks from March 2020 onwards, ‘Restrictions eased’ includes all diary weeks from July 2020 (when on‐trade premises re‐opened and restrictions on visiting other households were relaxed) onwards and ‘Some restrictions re‐introduced’ includes all diary weeks from October 2020 (when the tier system was introduced, and also covering the period of England’s second lockdown from November 2020) onwards. Results for the AR terms and seasonal AR terms included in each model are reported underneath the main results. L refers to number of lags, and these were selected following an iterative process involving autocorrelation function/autocorrelation function (ACF/PACF) plots and model fit statistics.

**TABLE 6:** Estimated impact of the introducing, easing and re‐introducing COVID‐19 lockdown restrictions on drinking occasion characteristics (who with, off‐trade location and start time) in England (step changes)

	Mean number of occasions per week (by who with)						Mean number of occasions per week (by off‐trade location)				Mean start time of first drinking occasion	
	(1) Solitary		(2) With family/partner		(3) With friends/colleagues		(4) Own home		(5) Someone else’s home		(6) Start time	
	B (95% CI)	*P*	B (95% CI)	*P*	B (95% CI)	*P*	B (95% CI)	*P*	B (95% CI)	*P*	B (95% CI)	*P*
Initial lockdown	−0.01	0.845	0.06	0.253	−0.33	< 0.001	0.27	< 0.001	−0.07	< 0.001	0.40	< 0.001
	(−0.12, 0.10)		(−0.04, 0.15)		(−0.37, −0.29)		(0.21, 0.34)		(−0.10, −		(0.29, 0.51)	
Restrictions eased	−0.02 (−0.14, 0.10)	0.730	0.04 (−0.20, 0.27)	0.77	0.24 (0.15, 0.33)	< 0.001	−0.05 (−0.21, 0.11)	0.525	0.04)	< 0.001	−0.28 (−0.41, −0.14)	< 0.001
Some restrictions re‐introduced	0.08 (−0.00, 0.16)	0.051	−0.09 (−0.38, 0.19)	0.537	−0.14 (−0.20, −0.07)	< 0.001	−0.00 (−0.16, 0.15)	0.987	0.07 (0.03, 0.10)	0.247	0.28 (0.12, 0.44)	0.001
Constant	0.42 (0.17, 0.68)	0.001	4.64 (4.04, 5.24)	< 0.001	2.25 (1.49)	< 0.001	4.00	< 0.001	−0.04 (−0.10, 0.03)	< 0.001	19.31 (18.76, 19.87)	< 0.001
AR terms	L1: 0.21 (0.04, 0.39)	0.017	L1: 0.25 (0.07, 0.42)	0.005	L2: 0.32 (0.15, 0.49)	< 0.001	(3.37, 4.62) L1: 0.20 (0.05, 0.35)	0.009	0.98 (0.81, 1.16)	0.016	L17: −0.14	0.163
	L11: 0.21 (0.04, 0.39)	0.018	L3: 0.21 (0.04, 0.39)	0.015	L3: 0.31 (0.13, 0.49)	0.001	L6: 0.18 (−0.01, 0.37) L11: 0.17 (−0.03, 0.39)	0.059 0.101	L4: 0.23 (0.04, 0.42) L22: −0.20 (−0.46, 0.05)	0.114	(−0.34, 0.06)	
Seasonal (12) AR terms	L1: 0.25 (0.04, 0.46)	0.017	L1: 0.31 (0.11, 0.51)	0.002	L1: 0.21 (< 0.001, 0.42) L2: 0.07 (−0.18, 0.32) L3: 0.34 (0.10, 0.59)	0.050 0.577 0.006	L1: 0.19 (−0.03, 0.42)	0.095	L1: 0.56 (0.38, 0.73)	< 0.001	L1: 0.52 (0.35, 0.69)	< 0.001

Estimates are adjusted for autocorrelation, seasonality and trend. B = coefficient, 95% confidence intervals (CI) and *P* = *p*‐value. ‘Initial lockdown’ includes all diary weeks from March 2020 onwards, ‘Restrictions eased’ includes all diary weeks from July 2020 (when on‐trade premises re‐opened and restrictions on visiting other households were relaxed) onwards and ‘Some restrictions re‐introduced’ includes all diary weeks from October 2020 (when the tier system was introduced, and also covering the period of England’s second lockdown from November 2020) onwards. Results for the AR terms and seasonal AR terms included in each model are reported underneath the main results. L refers to number of lags, and these were selected following an iterative process involving autocorrelation function/autocorrelation function (ACF/PACF) plots and model fit statistics.

### Changes in average weekly consumption

Figure 1 highlights that, following the initial March 2020 lockdown, mean off‐trade units per week increased and mean on‐trade units per week decreased. These changes largely persisted when restrictions were eased/re‐introduced, with off‐trade consumption mainly remaining above the 2016–19 average and on‐trade consumption below the 2016–19 average throughout the remainder of 2020. Total consumption was below the 2016–19 average when lockdown restrictions were in place, particularly during November–December, when consumption is traditionally high leading up to Christmas.

In Scotland, the ITS analysis suggests that the initial March 2020 lockdown was associated with a 2.32 [95% confidence interval (CI) = 0.61, 4.02] increase in mean off‐trade units per week and a −2.84 (95% CI = −3.63, −2.06) decrease in mean on‐trade units per week. The consequence of these off‐trade/on‐trade changes was a −0.84 (95% CI = −6.76, 5.09) decrease in total units per week, but this was not statistically significant. Subsequently, the July 2020 easing of restrictions was associated with a 1.33 (95% CI = 0.05, 2.62) increase in on‐trade units per week but no further statistically significant off‐trade/total consumption changes. The October 2020 re‐introduction of some restrictions in Scotland was not associated with any further statistically significant changes.

Results for England were broadly similar to Scotland. The initial March 2020 lockdown was associated with a 1.18 (95% CI = 0.65, 1.70) increase in mean off‐trade units per week and a −2.53 (95% CI = −2.86, −2.20) decrease in mean on‐trade units per week. As restrictions were subsequently eased then re‐introduced there were no further statistically significant off‐trade changes, but mean on‐trade units per week increased by 1.37 (95% CI = 0.82, 1.91) before decreasing by −0.73 (95% CI = −1.19, −0.26).

### Changes in other alcohol consumption measures

The results for the secondary consumption measures were largely consistent with the primary analysis. In the off‐trade, the ITS analysis suggests a statistically significant increase in heavy drinking occasions per week, drinking days per week and (in England only) proportion of individuals drinking > 14 units per week following the initial March 2020 lockdown, and these changes persisted as restrictions were eased/re‐introduced. In the on‐trade, there were decreases in drinking throughout all consumption measures following the initial lockdown. These mainly increased again when restrictions were eased (although not by enough to offset previous reductions). When restrictions were re‐introduced, i.e. the period when England had stricter rules than Scotland under its second national lockdown, on‐trade consumption again statistically significantly decreased in England, but not Scotland.

### Changes in drinking occasion characteristics

In Scotland, the initial March 2020 lockdown was associated with a 0.08 (95% CI = 0.02, 0.14) increase in the mean number of solitary drinking occasions per week. It was also associated with a −0.32 (95% CI = −0.45, −0.20) decrease in the mean number of occasions per week with friends/colleagues and a 0.38 (95% CI = 0.25, 0.51) increase in the mean number of occasions per week in respondents’ own homes. Finally, the initial March 2020 lockdown was associated with a −0.08 (95% CI = −0.14, −0.02) decrease in the mean number of occasions per week in someone else’s home, while the mean start time shifted to later in the day by 0.59 (95% CI = 0.16, 1.02) of an hour, i.e. 35.4 minutes. There were no further statistically significant changes as restrictions were subsequently eased/re‐introduced.

In England, there were no statistically significant changes in solitary drinking following changes to lockdown restrictions. There were, however, changes in other characteristics. Following the initial March 2020 lockdown start‐times shifted to later in the day and there were fewer occasions with friends/colleagues and in someone else’s home. These changes largely reverted to previous levels as restrictions were eased (before changing again as they were re‐introduced). The mean number of occasions per week in respondents’ own homes increased following the initial March 2020 lockdown. This persisted as restrictions were eased/re‐introduced.

### Model assumptions and sensitivity analyses

Model assumptions (i.e. normality of residuals and freedom from autocorrelation) were tested using kernel density plots and portmanteau statistics. These are provided in Supporting information, Appendices D and E. In addition, two sensitivity analyses were carried out to address potential concerns related to the main analysis. First, one population‐level policy introduced during the analysis period (in May 2018) in Scotland was minimum unit pricing (MUP). To ensure that this did not influence the results, the ITS analysis of consumption measures in Scotland was repeated controlling for MUP’s introduction. The results (provided in Supporting information, Appendix F) suggest that MUP did not substantively affect the model coefficients or *P*‐values. Secondly, to ensure that the results were not affected by the units per week capping process, the primary analysis was repeated using uncapped data. The results (provided in Supporting information, Appendix G) suggest that capping also did not substantively affect the model coefficients or *P*‐values.

## DISCUSSION

This study used Kantar Alcovision data to examine how alcohol consumption and drinking occasion characteristics changed during three periods of changes in lockdown restrictions in Scotland/England in 2020. The results suggest that, while total consumption remained fairly stable, this masked significant changes in how people were drinking in terms of off‐trade versus on‐trade consumption and the characteristics of drinking occasions.

Off‐trade consumption increased following the initial March 2020 lockdown in Scotland and England, and remained persistently higher than in previous years throughout the remainder of 2020 as restrictions were eased/re‐introduced. Meanwhile, on‐trade consumption decreased following the initial March 2020 lockdown and, despite increasing again when restrictions were relaxed in July, remained lower than in previous years. This is probably because some premises (e.g. nightclubs and live music venues) remained closed, while those that were open were operating at reduced capacity. Some people who previously drank in on‐trade premises may also have stayed away due to fears of catching the virus. As restrictions began to be re‐introduced from October 2020, on‐trade alcohol consumption decreased statistically significantly in England but not Scotland, which reflects England’s harsher restrictions during its second lockdown. Overall, these findings back up previous UK research [11, 12, 13, 16] in suggesting that, in general, when access to on‐trade drinking was restricted people tended to substitute most of their previous on‐trade drinking with greater off‐trade consumption. The effect sizes observed are fairly large. For example, to put the Scotland initial lockdown coefficients into context, mean consumption in 2016–19 in Scotland was 10.9 off‐trade units per week and 4.7 on‐trade units per week. Therefore, the initial lockdown corresponded to a 21.3% increase in off‐trade consumption and a 60.4% decrease in on‐trade consumption. This is broadly in line with effect sizes observed in existing research on the impact of lockdown restrictions on UK consumption [11, 12, 13, 16].

This study also highlights changes to drinking occasion characteristics in 2020. Traditionally, UK drinking practices involve a diverse range of occasion types, occurring with a variety of different companions and in a variety of different contexts/temporalities/locations [27]. Lockdown measures affect this by restricting where people can drink and their ability to socialize. Moreover, it has been argued that they can lead to temporal changes to drinking occasions by altering peoples’ routines and the amount of stress/boredom they face [28]. This study suggests that when under lockdown restrictions drinkers substituted occasions in someone else’s home with more occasions in their own home, while there were also fewer occasions with friends/colleagues. Lockdown restrictions were also associated with spikes in solitary drinking occasions in Scotland, but not England. This may reflect Scotland’s disproportionately high number of one‐person households [29]. Finally, the results suggest that drinking occasions shifted to later in the day under lockdown restrictions, which goes against concerns raised early in the pandemic that home‐working and job retention schemes would increase daytime drinking due to less structured days and a disconnection from employers. One explanation for this finding is that occasions starting earlier are traditionally associated with longer drinking occasions, often involving groups of friends and a mixture of on‐trade/off‐trade locations [30]. Lockdown restrictions prevent this type of occasion from taking place.

The key strength of this study is that, while many surveys changed their previously established methods during the pandemic [22], Alcovision continued its pre‐COVID data collection methods unchanged. Furthermore, while existing studies have tended to only have data on the early stages of the pandemic and focus upon alcohol consumption only, this study included monthly data up to the end of 2020, and provides insight into drinking occasion characteristics.

However, there are some limitations to note. First, like many large‐scale alcohol surveys [31] Alcovision relies upon quota sampling from an on‐line panel rather than random sampling. This has known limitations relating to selection bias [32], although we used a ‘raking’ technique to increase representativeness. Secondly, ITS analysis works best with a substantial amount of pre‐ and post‐intervention data [33]. As COVID‐19 is still a recent phenomenon, our analysis is limited by its relatively small post‐intervention sample size, although our large pre‐intervention sample allows models to account for pre‐existing trends. Thirdly, the three intervention points used in the analysis were based on calendar months, so do not perfectly align with restriction changes occurring mid‐month. This means that the modelling may underestimate the impact of changing restrictions. Fourthly, on‐trade consumption did not fall to zero when premises were closed during lockdowns. This phenomenon has also been seen in Australian survey data [34]. It may occur due to people misreporting take‐away alcohol as on‐trade consumption, illegal on‐trade consumption taking place or hotels legally selling alcohol to guests residing there for essential work [12].

To conclude, COVID‐19 lockdown restrictions represent a substantial intervention, with important implications for drinking practices. This study has highlighted that restrictions in Scotland and England were associated with statistically significant changes in off‐trade/on‐trade alcohol consumption and to drinking occasion characteristics, with many of these changes persisting in periods of greater/lesser restrictions. Looking ahead, it remains unclear what the long‐term consequences of this will be. On‐trade consumption is likely to move closer to pre‐pandemic levels as on‐trade premises return to operating at full capacity, and people become less afraid of indoor public spaces. However, one concern is the observed increase in ‘home drinking’. While ‘home drinking’ is currently a relatively under‐researched topic [7, 35], its increase is likely to have contributed to the high levels of alcohol‐related harm during the pandemic [16]. There is a need to monitor this further in the future to ascertain whether ‘home drinking’ habits picked up during 2020 become a ‘new norm’ within people’s drinking behaviour.

## DECLARATION OF INTERESTS

None.

## AUTHOR CONTRIBUTIONS

**Iain Hardie:** Conceptualization; formal analysis; methodology; writing ‐ original draft. **Abigail Stevely:** Conceptualization; formal analysis; methodology; writing ‐ review & editing. **Alessandro Sasso:** Data curation; writing ‐ review & editing. **Petra Meier:** Conceptualization; funding acquisition; project administration; writing ‐ review & editing. **John Holmes:** Conceptualization; funding acquisition; project administration; writing ‐ review & editing.

## Supporting information


**Data S1.** Supporting Information

**Data S2.** Supporting Information

**Figure S7.** Mean proportion drinking >14 units per week in Scotland (vertical lines from left to right = months where lockdown restrictions were introduced, eased and reintroduced)

**Figure S8.** Mean heavy drinking occasions per week in Scotland (vertical lines from left to right = months where lockdown restrictions were introduced, eased and reintroduced)

**Figure S9.** Mean drinking days per week in Scotland (vertical lines from left to right = months where lockdown restrictions were introduced, eased and reintroduced)

**Figure S10.** Mean drinking occasions per week by who with in Scotland (vertical lines from left to right = months where lockdown restrictions were introduced, eased and reintroduced)

**Figure S11.** Mean drinking occasions per week by off‐trade location in Scotland (vertical lines from left to right = months where lockdown restrictions were introduced, eased and reintroduced)

**Figure S12.** Mean start time of first drinking occasion per day in Scotland (vertical lines from left to right = months where lockdown restrictions were introduced, eased and reintroduced)

**Figure S13.** Mean proportion drinking >14 units per week in England (vertical lines from left to right = months where lockdown restrictions were introduced, eased and reintroduced)

**Figure S14.** Mean heavy drinking occasions per week in England (vertical lines from left to right = months where lockdown restrictions were introduced, eased and reintroduced)

**Figure S15.** Mean drinking days per week in England (vertical lines from left to right = months where lockdown restrictions were introduced, eased and reintroduced)

**Figure S16.** Mean drinking occasions per week by who with in England (vertical lines from left to right = months where lockdown restrictions were introduced, eased and reintroduced)

**Figure S17.** Mean drinking occasions per week by off‐trade location in England (vertical lines from left to right = months where lockdown restrictions were introduced, eased and reintroduced)

**Figure S18.** Mean start time of first drinking occasion per day in England (vertical lines from left to right = months where lockdown restrictions were introduced, eased and reintroduced)

**Figure S19.** Mean units per week in Scotland (vertical lines from left to right = months where lockdown restrictions were introduced, eased and reintroduced)

**Figure S20.** Mean Proportion Drinking >14 Units per week in Scotland (vertical lines from left to right = months where lockdown restrictions were introduced, eased and reintroduced)

**Figure S21.** Mean heavy drinking occasions per week in Scotland (vertical lines from left to right = months where lockdown restrictions were introduced, eased and reintroduced)

**Figure S22.** Mean drinking days per week in Scotland (vertical lines from left to right = months where lockdown restrictions were introduced, eased and reintroduced)

**Figure S23.** Mean drinking occasions per week by who with in Scotland (vertical lines from left to right = months where lockdown restrictions were introduced, eased and reintroduced)

**Figure S24.** Mean drinking occasions per week by off‐trade location in Scotland (vertical lines from left to right = months where lockdown restrictions were introduced, eased and reintroduced)

**Figure S25.** Mean start time of first drinking occasion per day in Scotland (vertical lines from left to right = months where lockdown restrictions were introduced, eased and reintroduced)

**Figure S26.** Mean units per week in England (vertical lines from left to right = months where lockdown restrictions were introduced, eased and reintroduced)

**Figure S27.** Mean Proportion Drinking >14 Units per week in England (vertical lines from left to right = months where lockdown restrictions were introduced, eased and reintroduced)

**Figure S28.** Mean heavy drinking occasions per week in England (vertical lines from left to right = months where lockdown restrictions were introduced, eased and reintroduced)

**Figure S29.** Mean drinking days per week in England (vertical lines from left to right = months where lockdown restrictions were introduced, eased and reintroduced)

**Figure S30.** Mean drinking occasions per week by who with in England (vertical lines from left to right = months where lockdown restrictions were introduced, eased and reintroduced)

**Figure S31.** Mean drinking occasions per week by off‐trade location in England (vertical lines from left to right = months where lockdown restrictions were introduced, eased and reintroduced)

**Figure S32.** Mean start time of first drinking occasion per day in Scotland (vertical lines from left to right = months where lockdown restrictions were introduced, eased and reintroduced)

**Figure S33.** Mean Units per Week (Table 3) kernel density plots showing model residuals with normal density overlaid

**Figure S34.** Proportion of individuals drinking >14 units per week (Table 3) kernel density plots showing model residuals with normal density overlaid

**Figure S35.** Mean number of heavy drinking occasions per week (Table 3) kernel density plots showing model residuals with normal density overlaid

**Figure S36.** Mean number of drinking days per week (Table 3) kernel density plots showing model residuals with normal density overlaid

**Figure S37.** Mean number of solitary occasions per week (Table 5) kernel density plots showing model residuals with normal density overlaid

**Figure S38.** Mean number of occasions per week with family/partner (Table 5) kernel density plots showing model residuals with normal density overlaid

**Figure S39.** Mean number of occasions per week with friends/colleagues (Table 5) kernel density plots showing model residuals with normal density overlaid

**Figure S40.** Mean number of occasions per week in own home (Table 5) kernel density plots showing model residuals with normal density overlaid

**Figure S41.** Mean number of occasions per week in someone else's home (Table 5) kernel density plots showing model residuals with normal density overlaid

**Figure S42.** Mean start time of first drinking occasion (Table 5) kernel density plots showing model residuals with normal density overlaid

**Figure S43.** Mean Units per Week (Table 4) kernel density plots showing model residuals with normal density overlaid

**Figure S44.** Proportion of individuals drinking >14 units per week (Table 4) kernel density plots showing model residuals with normal density overlaid

**Figure S45.** Mean number of heavy drinking occasions per week (Table 4) kernel density plots showing model residuals with normal density overlaid

**Figure S46.** Mean number of drinking days per week (Table 4) kernel density plots showing model residuals with normal density overlaid

**Figure S47.** Mean number of solitary occasions per week (Table 6) kernel density plots showing model residuals with normal density overlaid

**Figure S48.** Mean number of occasions per week with family/partner (Table 6) kernel density plots showing model residuals with normal density overlaid

**Figure S49.** Mean number of occasions per week with friends/colleagues (Table 6) kernel density plots showing model residuals with normal density overlaid

**Figure S50.** Mean number of occasions per week in own home (Table 6) kernel density plots showing model residuals with normal density overlaid

**Figure S51.** Mean number of occasions per week in someone else's home (Table 6) kernel density plots showing model residuals with normal density overlaid

**Figure S52.** Mean start time of first drinking occasion (Table 6) kernel density plots showing model residuals with normal density overlaid

**Table S1.** Mean units per week (Table 3) portmanteau test checking residuals resemble white noise

**Table S2.** Proportion of individuals drinking >14 units per week (Table 3) portmanteau test checking residuals resemble white noise

**Table S3.** Mean number of heavy drinking occasions per week (Table 3) portmanteau test checking residuals resemble white noise

**Table S4.** Mean number of drinking days per week (Table 3) portmanteau test checking residuals resemble white noise

**Table S5.** Mean number of solitary occasions per week (Table 5) portmanteau test checking residuals resemble white noise

**Table S6.** Mean number of occasions per week with family/partner (Table 5) portmanteau test checking residuals resemble white noise

**Table S7.** Mean number of occasions per week with friends/colleagues (Table 5) portmanteau test checking residuals resemble white noise

**Table S8.** Mean number of occasions per week in own home (Table 5) portmanteau test checking residuals resemble white noise

**Table S9.** Mean number of occasions per week in someone else's home (Table 5) portmanteau test checking residuals resemble white noise

**Table S10.** Mean start time of first drinking occasion (Table 5) portmanteau test checking residuals resemble white noise

**Table S11.** Mean units per week (Table 4) portmanteau test checking residuals resemble white noise

**Table S12.** Proportion of individuals drinking >14 units per week (Table 4) portmanteau test checking residuals resemble white noise

**Table S13.** Mean number of heavy drinking occasions per week (Table 4) portmanteau test checking residuals resemble white noise

**Table S14.** Mean number of drinking days per week (Table 4) portmanteau test checking residuals resemble white noise

**Table S15.** Mean number of solitary occasions per week (Table 6) portmanteau test checking residuals resemble white noise

**Table S16.** Mean number of occasions per week with family/partner (Table 6) portmanteau test checking residuals resemble white noise

**Table S17.** Mean number of occasions per week with friends/colleagues (Table 6) portmanteau test checking residuals resemble white noise

**Table S18.** Mean number of occasions per week in own home (Table 6) portmanteau test checking residuals resemble white noise

**Table S19.** Mean number of occasions per week in someone else's home (Table 6) portmanteau test checking residuals resemble white noise

**Table S20.** Mean start time of first drinking occasion (Table 6) portmanteau test checking residuals resemble white noise

**Data S3.** Supporting Information

**Data S4.** Supporting Information
