# Supplementary material for: The impact of changes in COVID‐19 lockdown restrictions on alcohol consumption and drinking occasion characteristics in Scotland and England in 2020: an interrupted time‐series analysis
Source: Addiction. 2022 Feb 2;117(6):1622–39. doi: 10.1111/add.15794 (PMC9302640; doi:10.1111/add.15794)
Supplement: Supplementary file 1 — Data S1. Supporting Information [file ADD-117-1622-s007.docx]

# SUPPORTING INFORMATION APPENDIX A. Extra Detail on Methods

**Part 1: Details on ‘Raking’ Weighting Process**

- First, the weighting process applies a weight to individuals such that the weighted distribution of social grade matches the distribution in the UK Census.
- Subsequently, an algorithm readjusts the weighted distribution based on geographic region, age, and sex. The process is iterated repeatedly until the marginal distribution of all target variables has been matched with the UK Census data.

**Part 2: Details on Process for Calculating ABV**

- For each occasion, participants report the type and, where available, brand of alcohol consumed (e.g. sparkling white wine, Smirnoff vodka). Participants also provide the serving size (e.g. pint, small stubby bottle, large wine glass) and the number of servings.
- We combined this data with information on alcoholic strengths (ABV) to calculate the number of units consumed for each drink reported in the diary week.
- We obtained information on ABVs from reliable websites for specific brands (e.g. supermarket websites, brand websites or specialist beer websites). When reliable information on ABVs was not available online, we used assumptions about the average ABVs for each type of drink (vodka, still white wine, beer, etc.) taken from previous research literature or provided in personal communications by key informants.

**Part 3: Details on Process for Capping Units per Week**

- In previous analyses, we have identified that a small proportion of Alcovision participants report very high consumption values for particular drinks, occasions or days, or for the diary week as a whole. We have therefore developed and applied methods for capping the data to prevent misreported or extreme values from distorting findings.
- We first constrain the amount of alcohol reported for each single type of drink within an occasion (a row in the dataset) to a maximum of 40 units (the amount of alcohol in a litre bottle of whisky).
- We then constrain the amount of alcohol reported in each drinking occasion to a maximum of 40 units. This is done by removing the excess units proportionally from each of the drinks (rows) associated with that occasion.
- We then constrain the amount of alcohol reported per day to a maximum of 40 units. Again, this is done by removing the excess units proportionally from each of the drinking occasions within the same day, for each individual.
- Finally, individuals’ weekly alcohol consumption is calculated by summing all of the (capped) units reported during the week. Hence, maximum weekly consumption is constrained to 280 units (i.e. 7 x 40 units).
