# Supplementary material for: The impact of changes in COVID‐19 lockdown restrictions on alcohol consumption and drinking occasion characteristics in Scotland and England in 2020: an interrupted time‐series analysis
Source: Addiction. 2022 Feb 2;117(6):1622–39. doi: 10.1111/add.15794 (PMC9302640; doi:10.1111/add.15794)

# SUPPORTING INFORMATION APPENDIX B

**Figure S1.** Proportion drinking >14 units per week in Scotland and England in 2020 (with 2016-19 average for comparison)


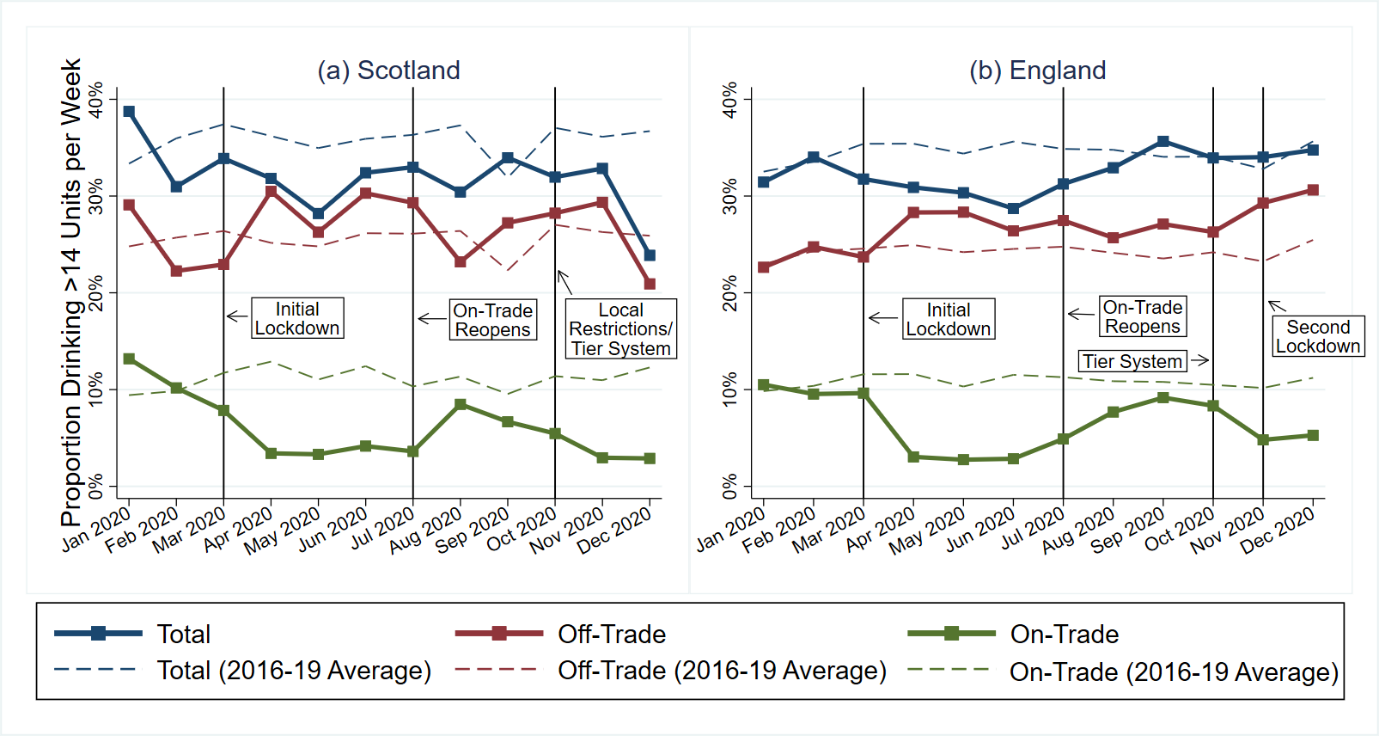


**Figure S2.** Mean heavy drinking occasions per week in Scotland and England in 2020 (with 2016-19 average for comparison)

**
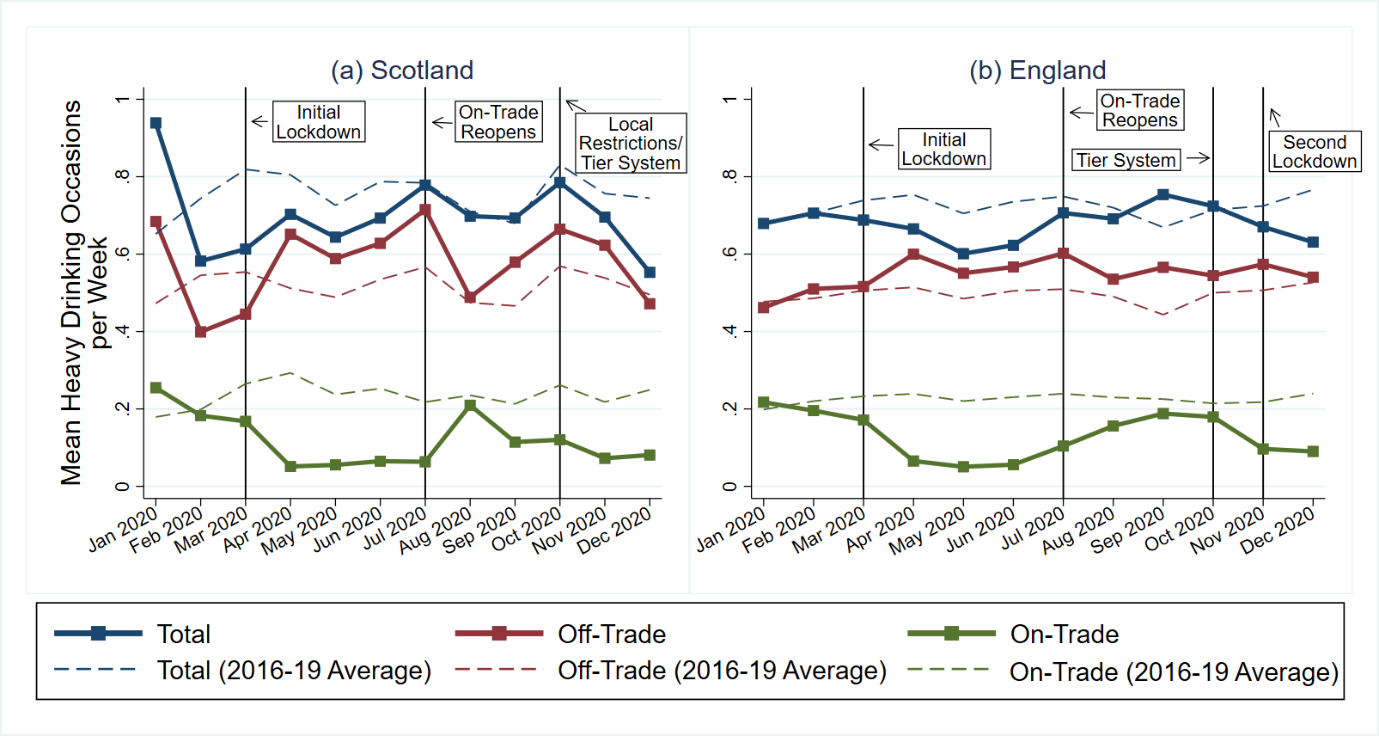
**

**Figure S3.** Mean drinking days per week in Scotland and England in 2020 (with 2016-19 average for comparison)


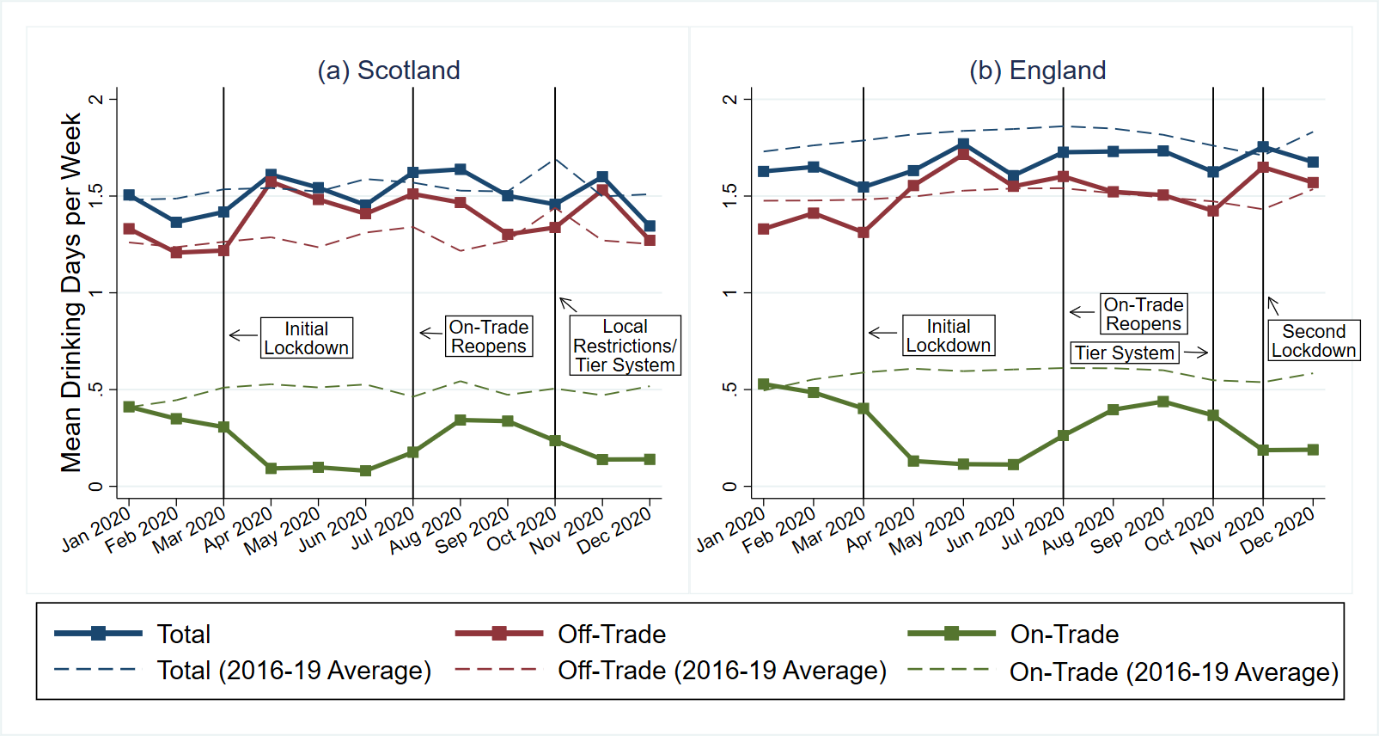


**Figure S4.** Mean drinking occasions per week (by who with) in Scotland and England in 2020 (with 2016-19 average for comparison)


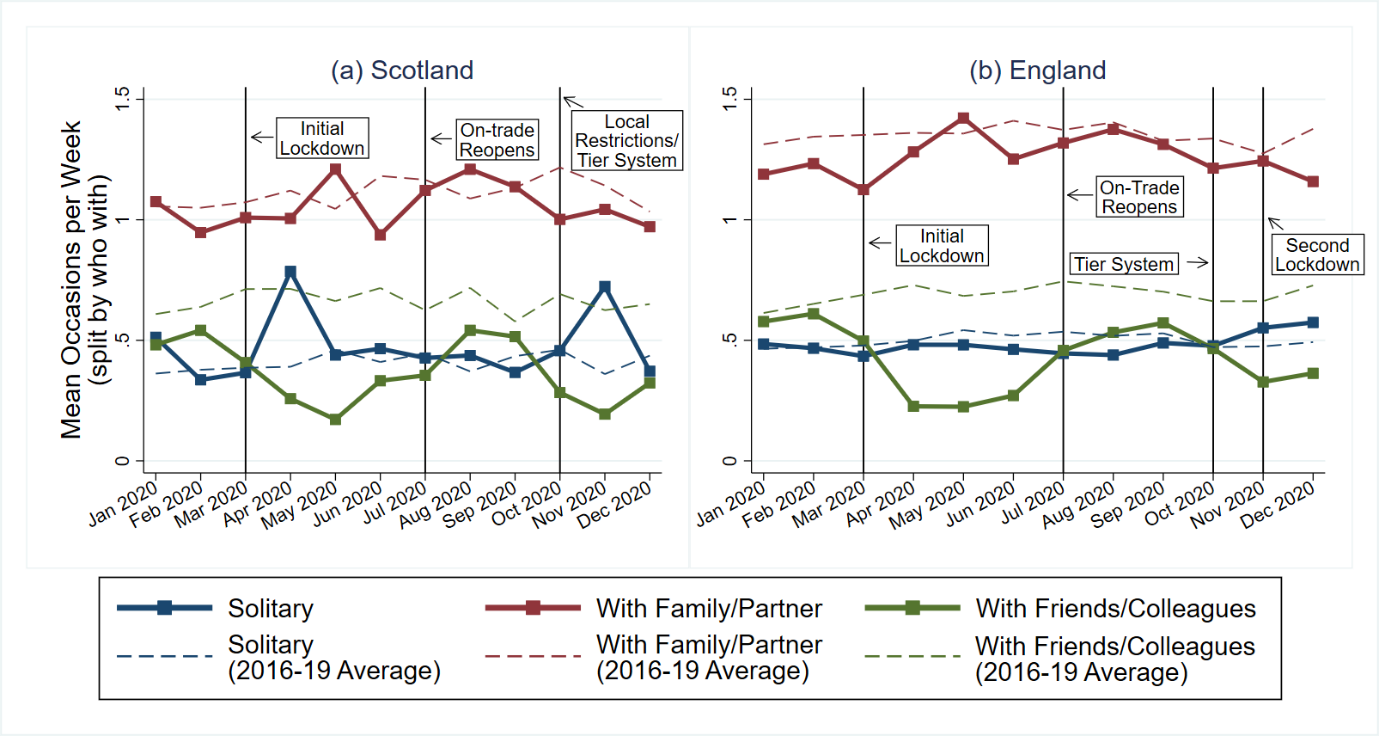


**Figure S5.** Mean drinking occasions per week (by off-trade location) in Scotland and England in 2020 (with 2016-19 average for comparison)


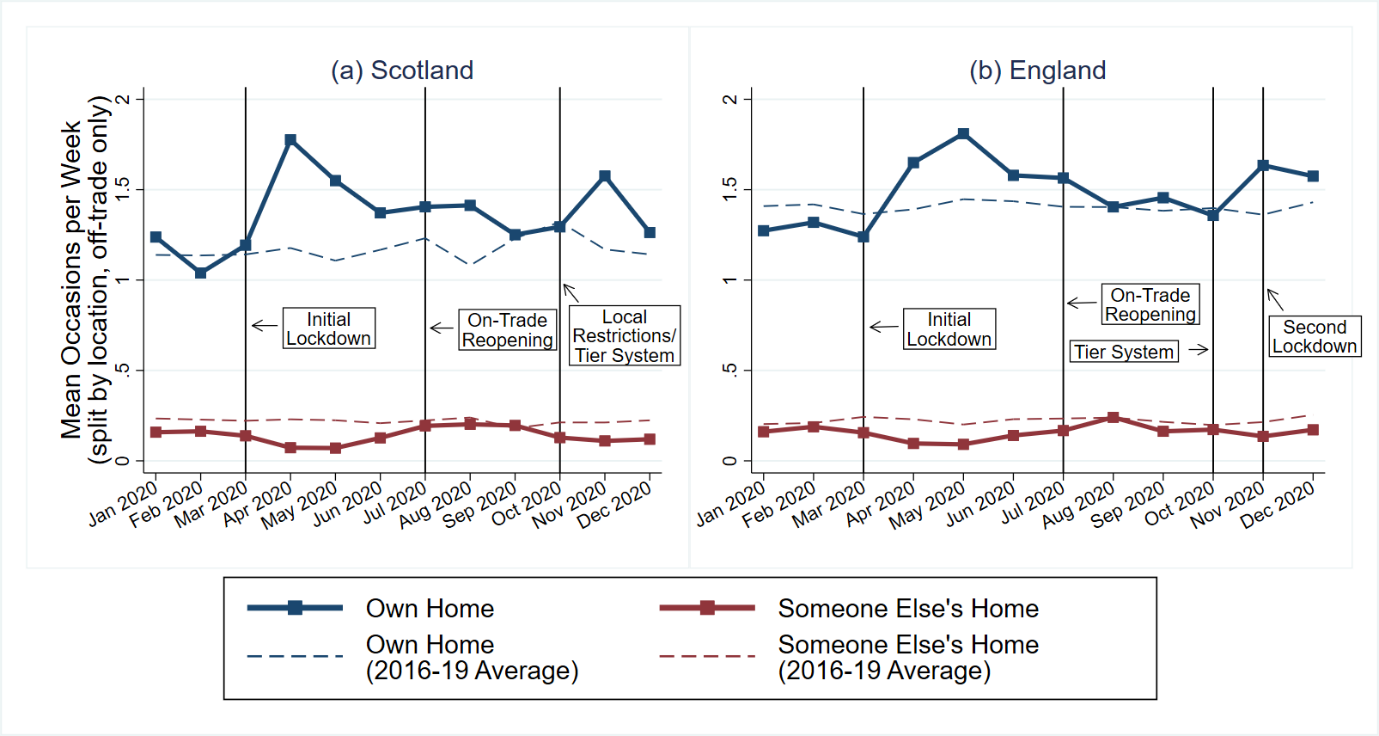


**Figure S6.** Mean start time of first drinking occasion per day in Scotland and England in 2020 (with 2016-19 average for comparison)


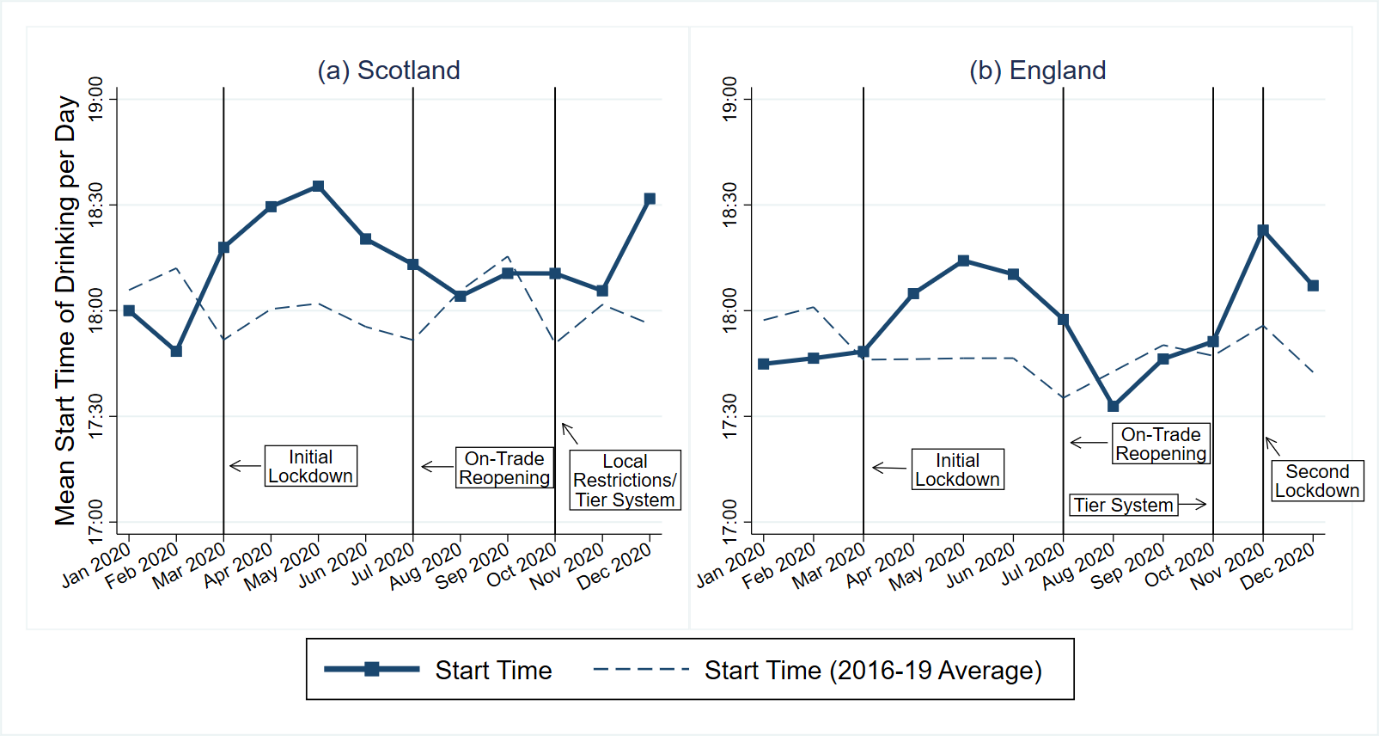

Supplement: Supplementary file 2 — Data S2. Supporting Information [file ADD-117-1622-s005.docx]
