# Supplementary material for: The impact of changes in COVID‐19 lockdown restrictions on alcohol consumption and drinking occasion characteristics in Scotland and England in 2020: an interrupted time‐series analysis
Source: Addiction. 2022 Feb 2;117(6):1622–39. doi: 10.1111/add.15794 (PMC9302640; doi:10.1111/add.15794)

# SUPPORTING INFORMATION APPENDIX C

## RAW MONTHLY TIME-SERIES AND ITS MODEL VALUES (SCOTLAND)

**Figure S7.** Mean proportion drinking >14 units per week in Scotland (vertical lines from left to right = months where lockdown restrictions were introduced, eased and reintroduced)


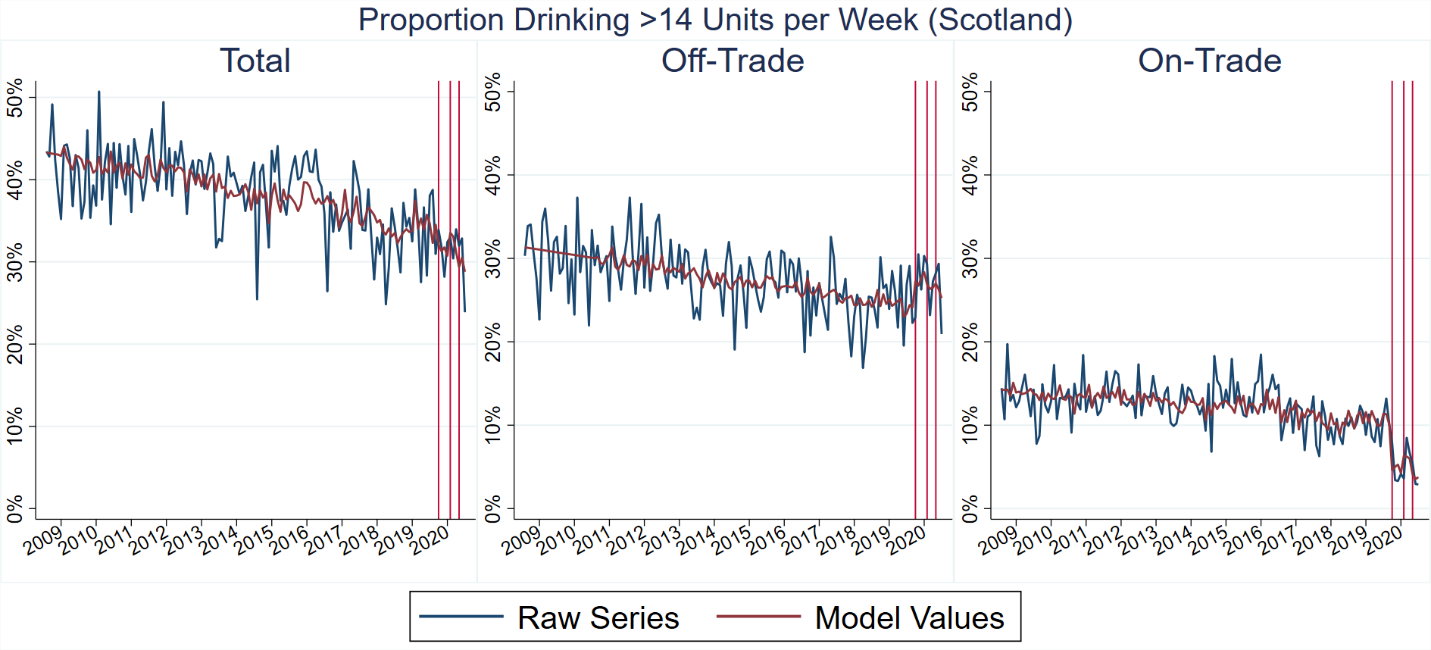


**Figure S8.** Mean heavy drinking occasions per week in Scotland (vertical lines from left to right = months where lockdown restrictions were introduced, eased and reintroduced)


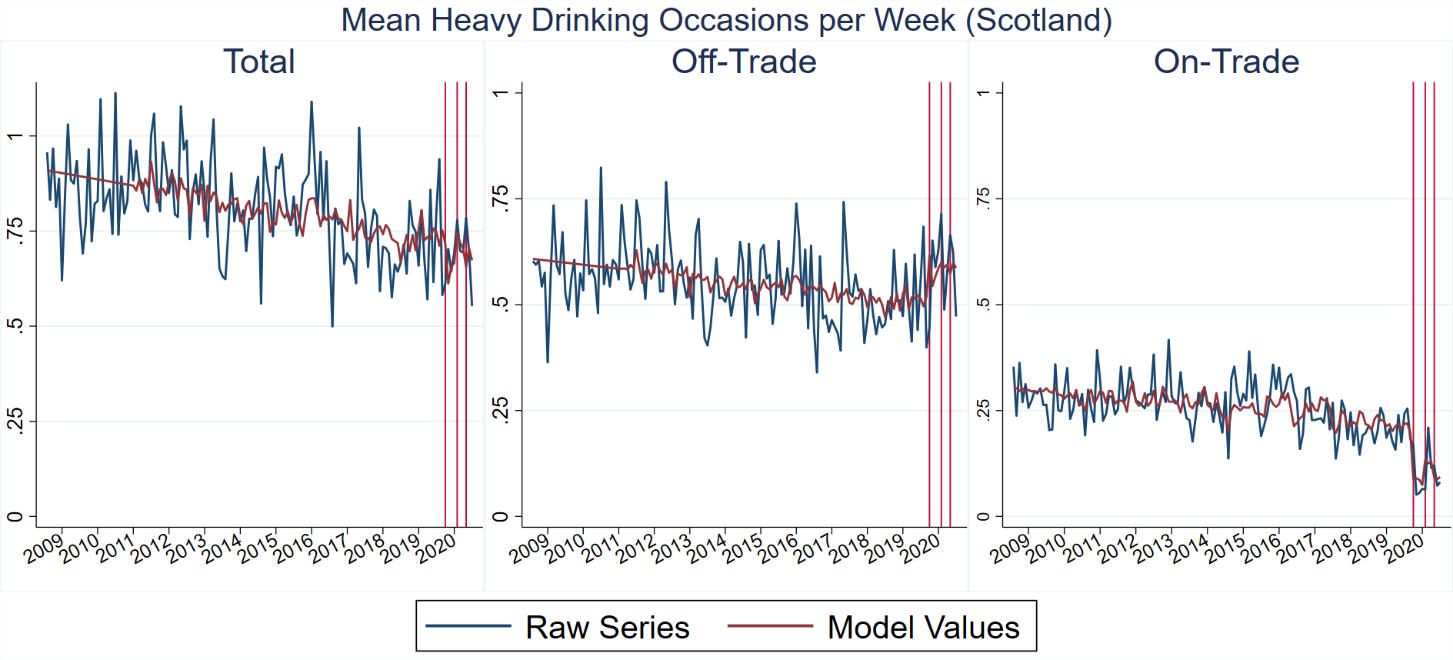


**Figure S9.** Mean drinking days per week in Scotland (vertical lines from left to right = months where lockdown restrictions were introduced, eased and reintroduced)


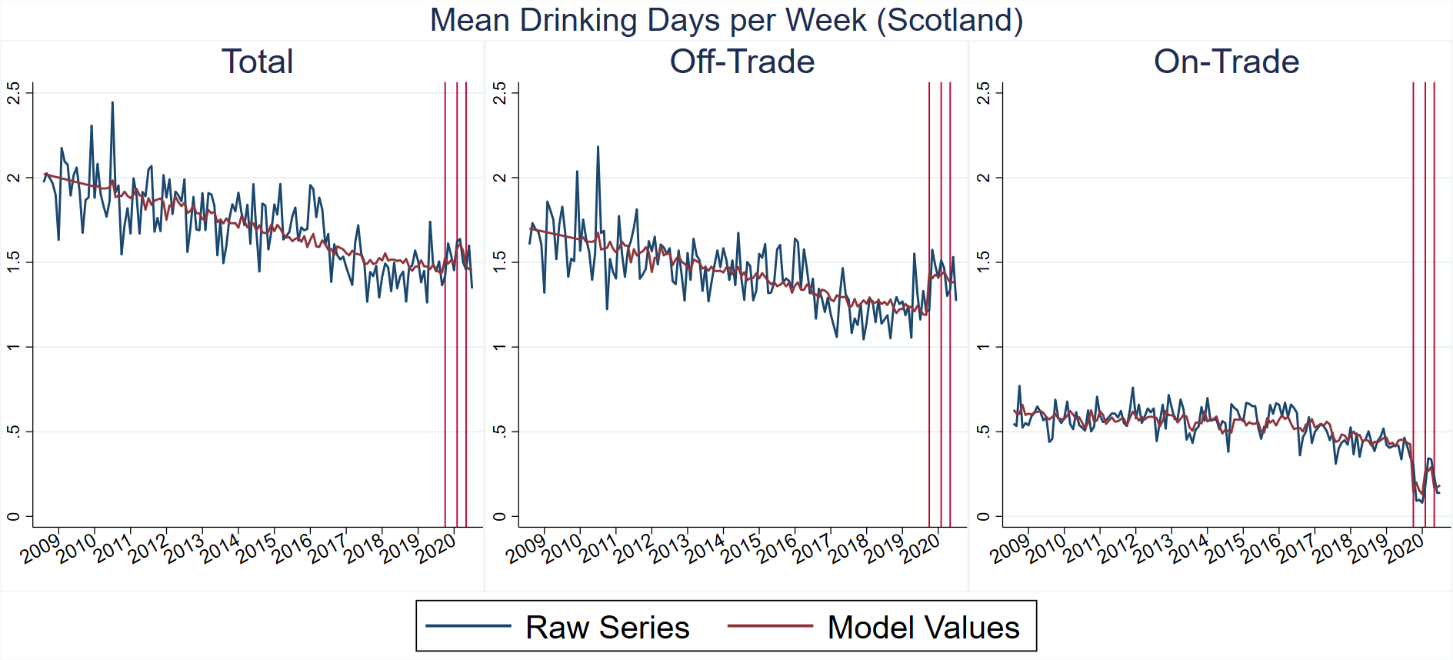


**Figure S10.** Mean drinking occasions per week by who with in Scotland (vertical lines from left to right = months where lockdown restrictions were introduced, eased and reintroduced)


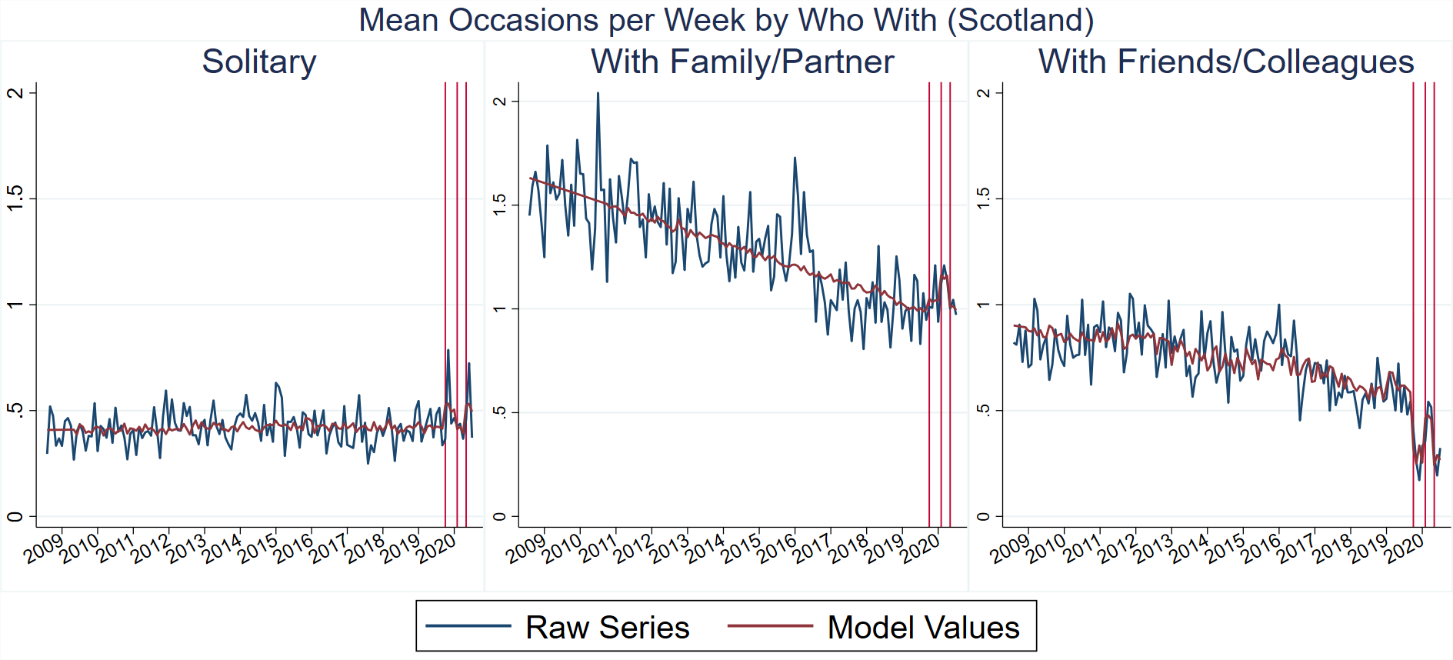


**Figure S11.** Mean drinking occasions per week by off-trade location in Scotland (vertical lines from left to right = months where lockdown restrictions were introduced, eased and reintroduced)


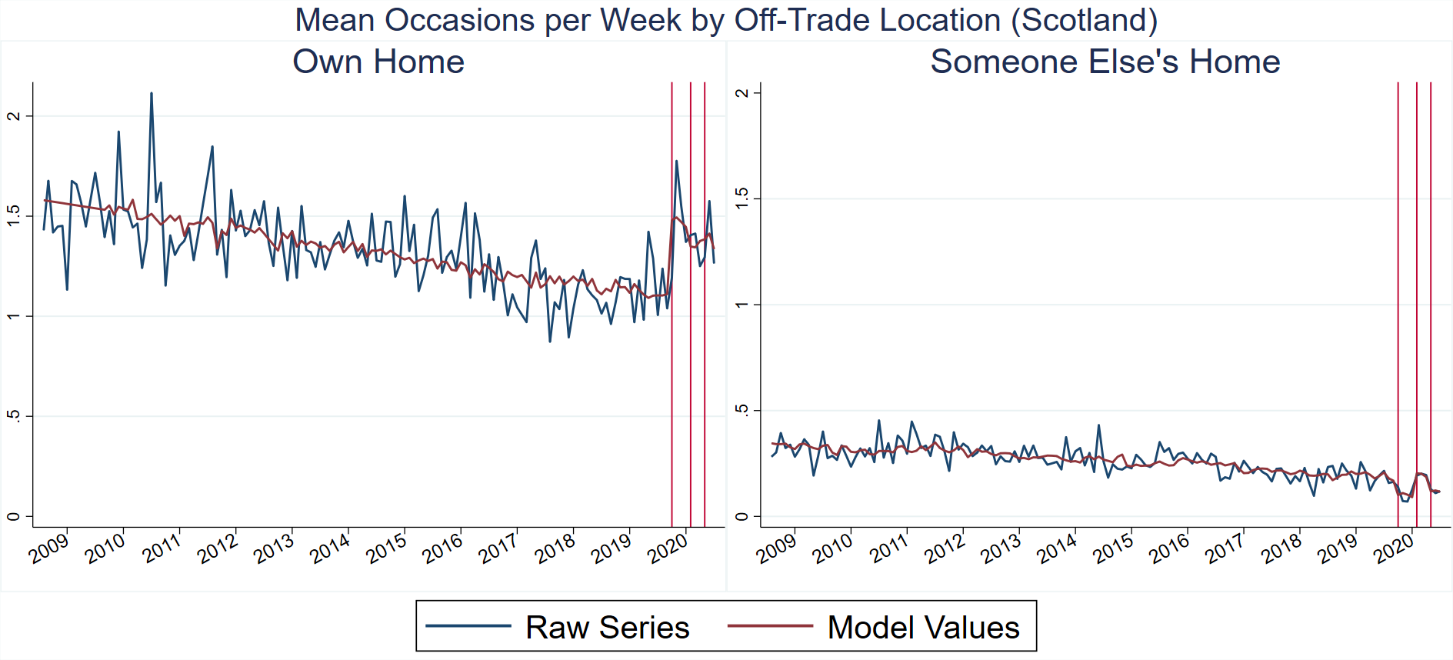


**Figure S12.** Mean start time of first drinking occasion per day in Scotland (vertical lines from left to right = months where lockdown restrictions were introduced, eased and reintroduced)


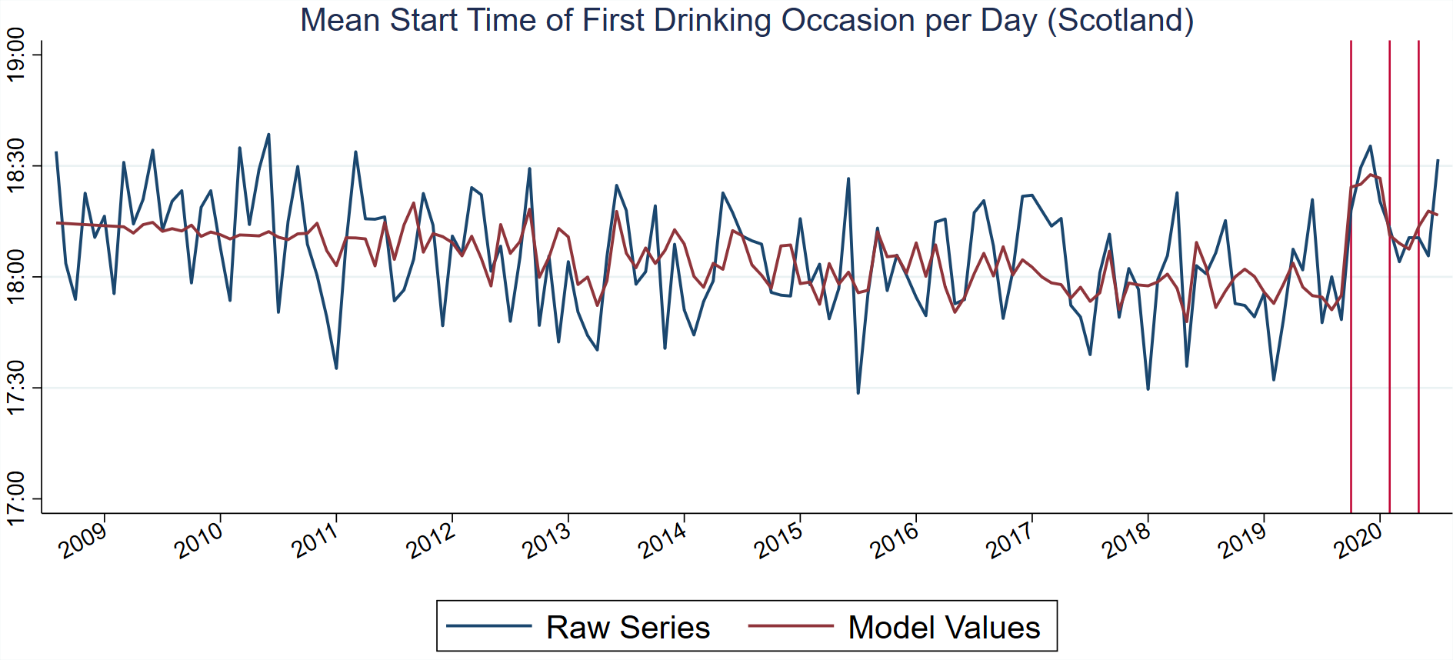


## RAW MONTHLY TIME-SERIES AND ITS MODEL VALUES (ENGLAND)

**Figure S13.** Mean proportion drinking >14 units per week in England (vertical lines from left to right = months where lockdown restrictions were introduced, eased and reintroduced)


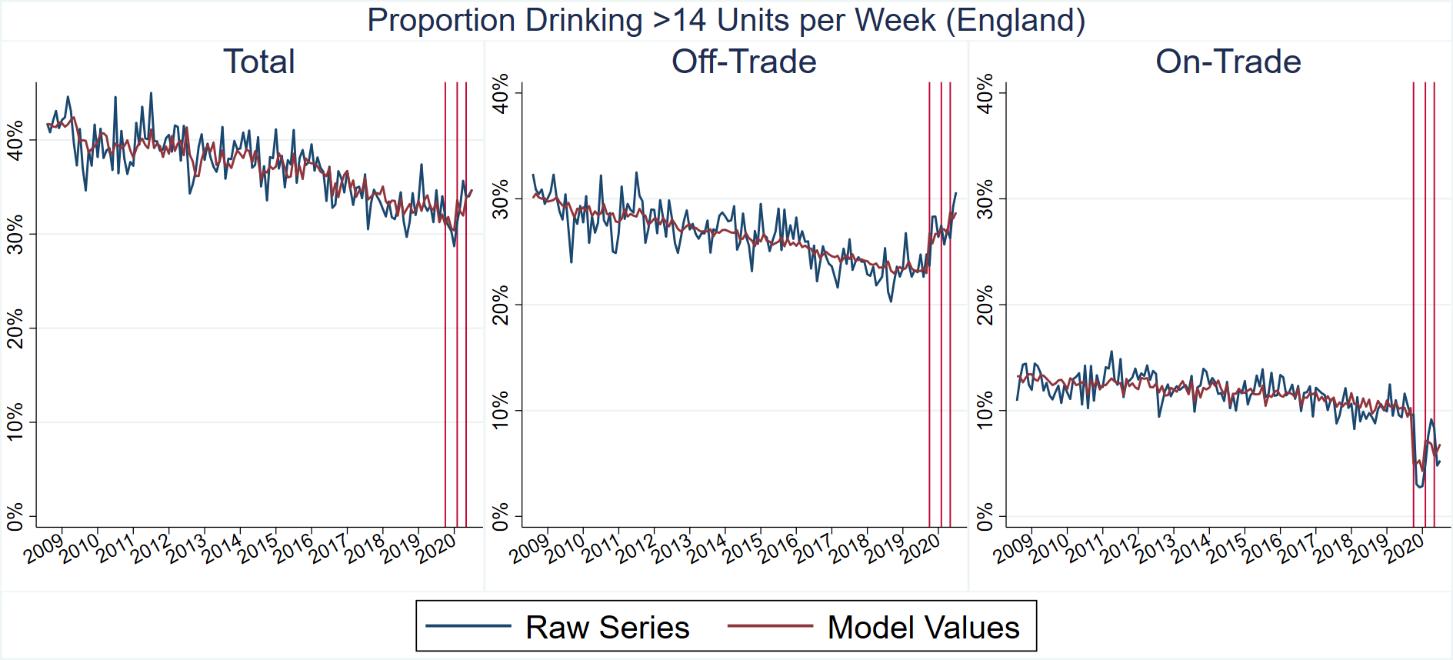


**Figure S14.** Mean heavy drinking occasions per week in England (vertical lines from left to right = months where lockdown restrictions were introduced, eased and reintroduced)


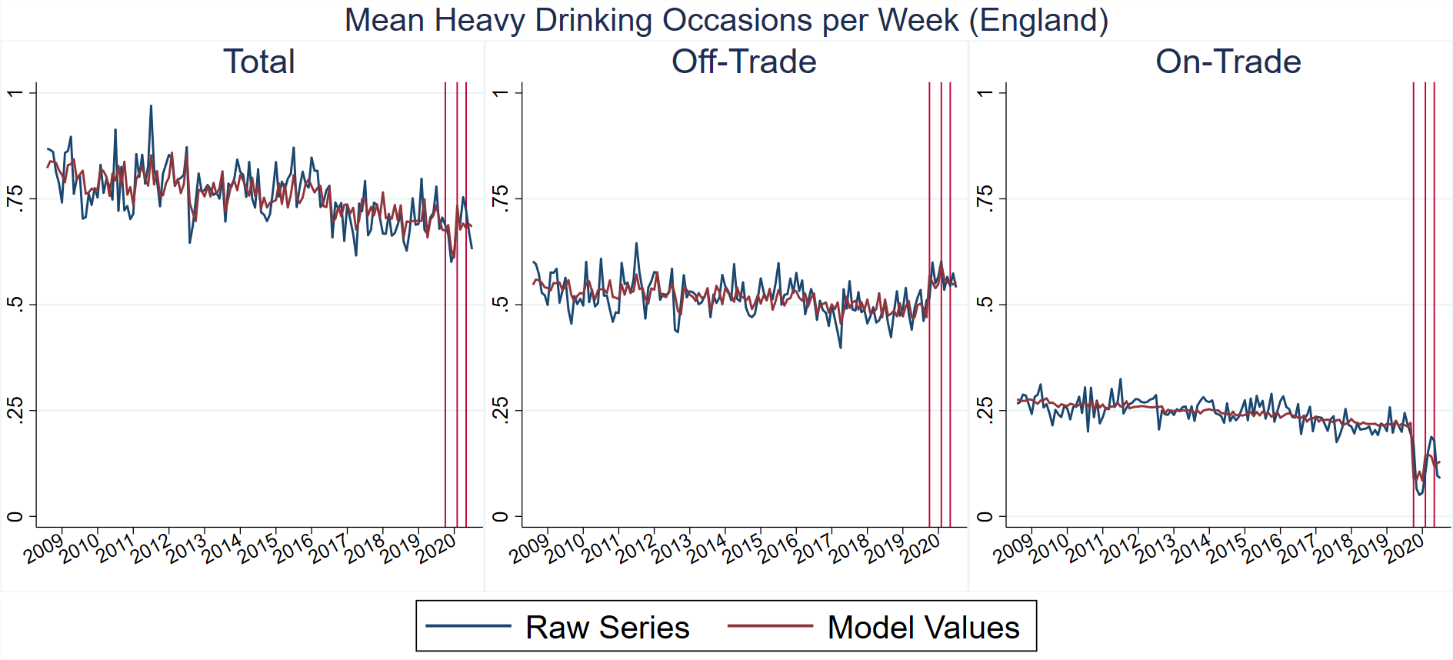


**Figure S15.** Mean drinking days per week in England (vertical lines from left to right = months where lockdown restrictions were introduced, eased and reintroduced)


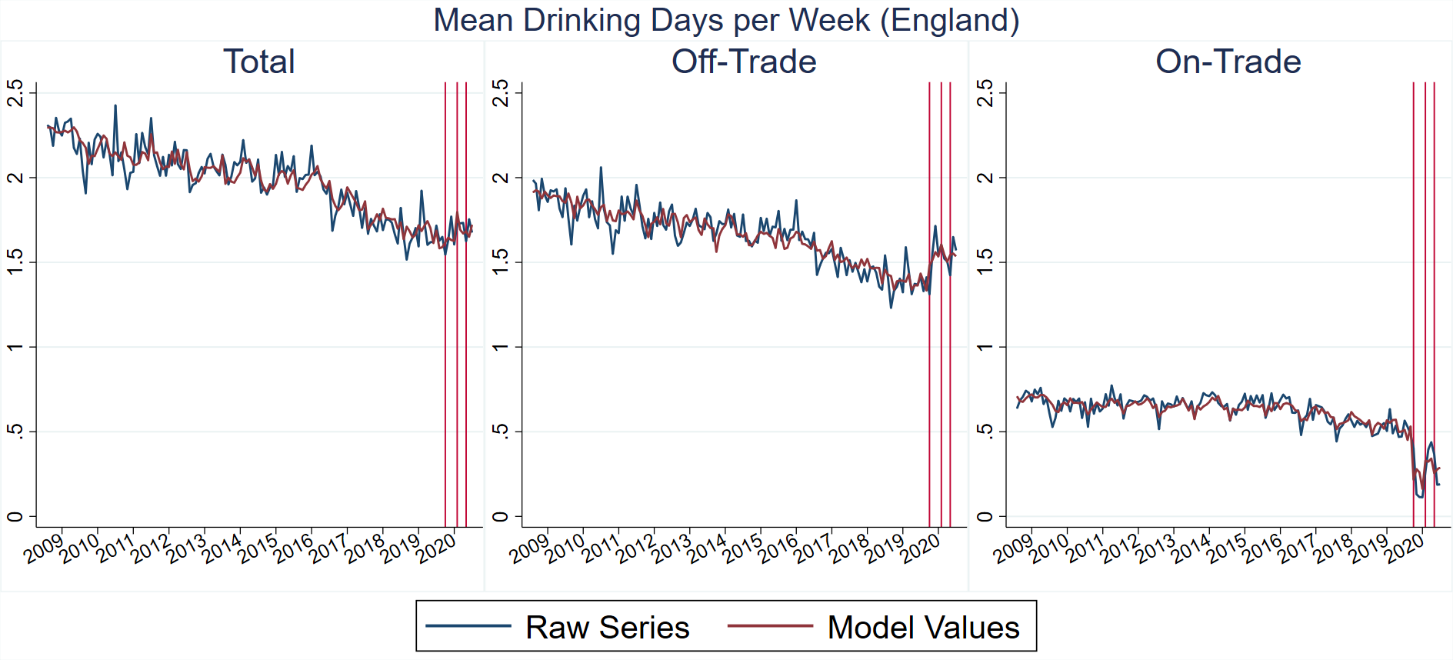


**Figure S16.** Mean drinking occasions per week by who with in England (vertical lines from left to right = months where lockdown restrictions were introduced, eased and reintroduced)


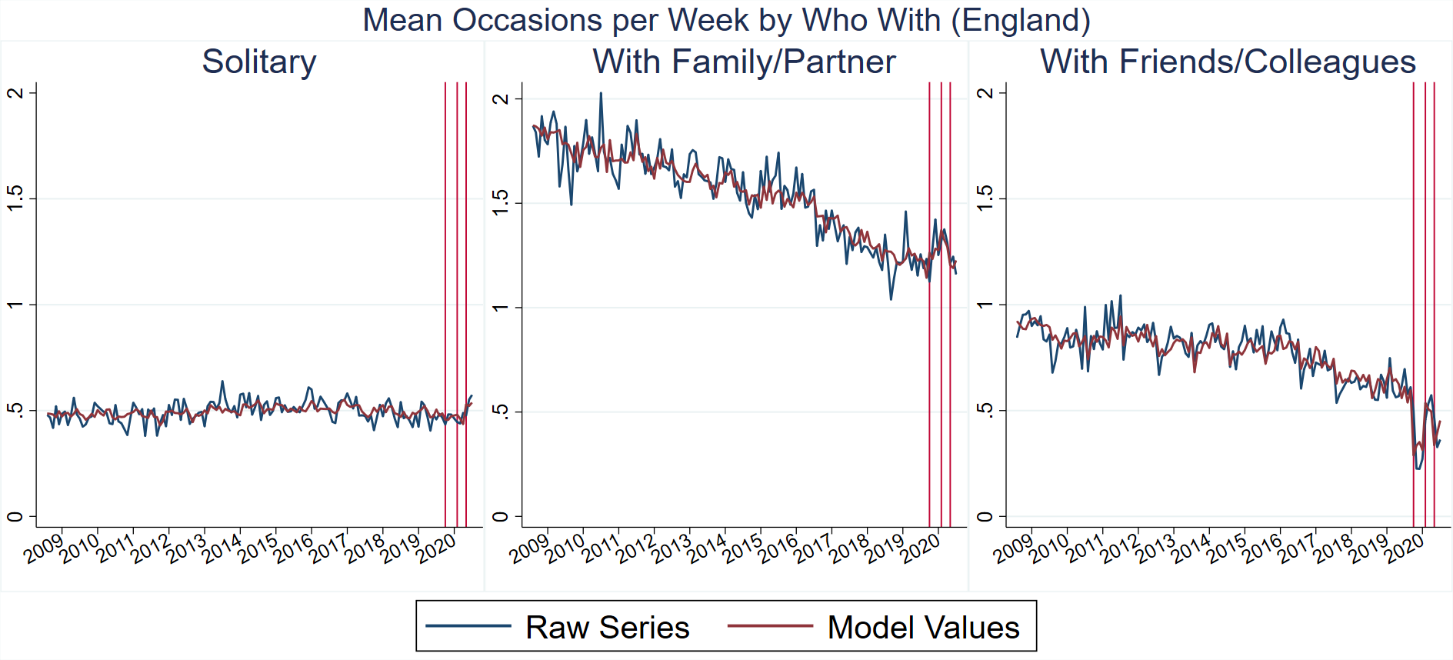


**Figure S17.** Mean drinking occasions per week by off-trade location in England (vertical lines from left to right = months where lockdown restrictions were introduced, eased and reintroduced)


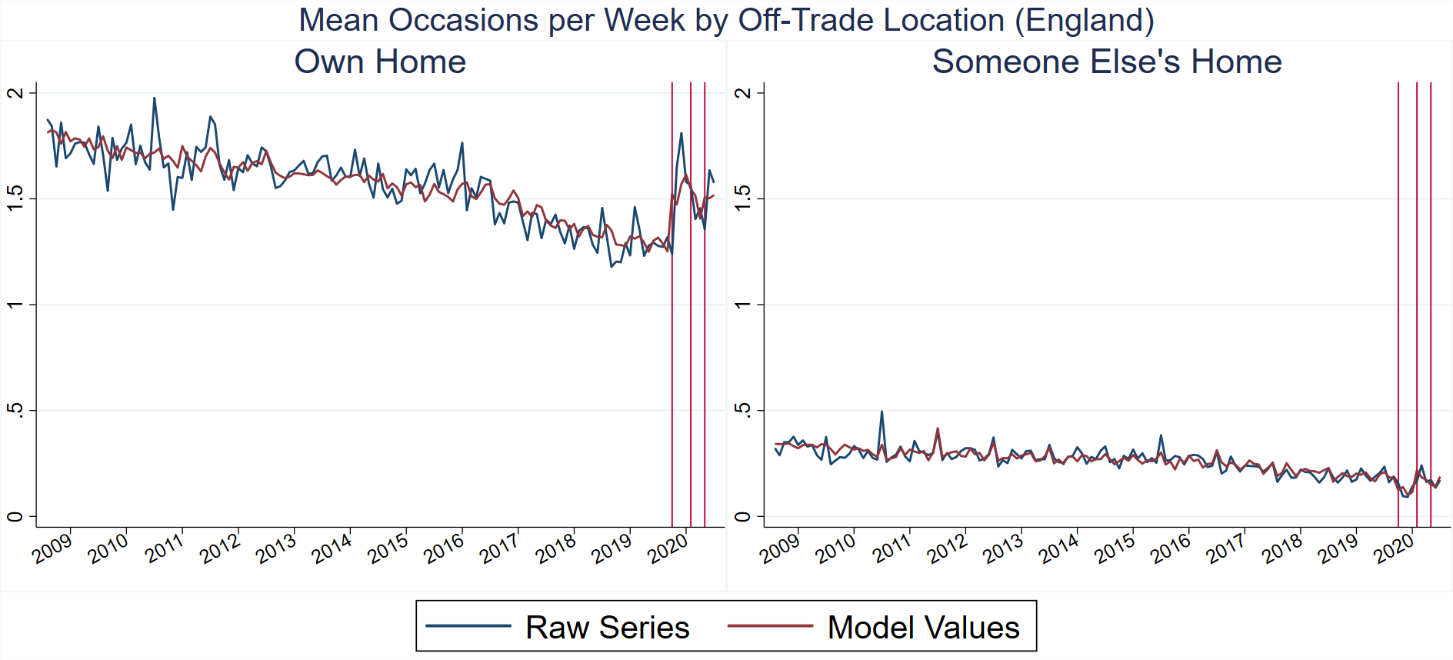


**Figure S18.** Mean start time of first drinking occasion per day in England (vertical lines from left to right = months where lockdown restrictions were introduced, eased and reintroduced)


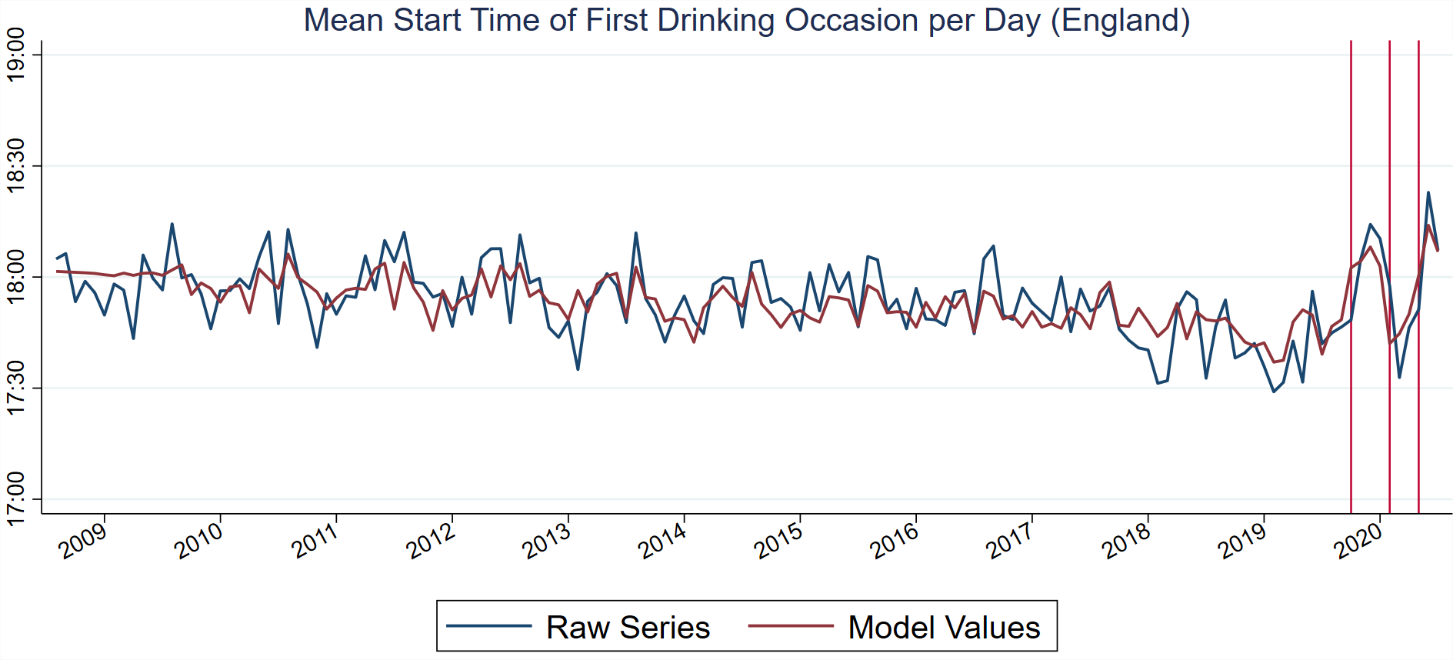


## RAW MONTHLY TIME-SERIES AND ITS MODEL VALUES (SCOTLAND, TRUNCATED VERSIONS)

**Figure S19.** Mean units per week in Scotland (vertical lines from left to right = months where lockdown restrictions were introduced, eased and reintroduced)


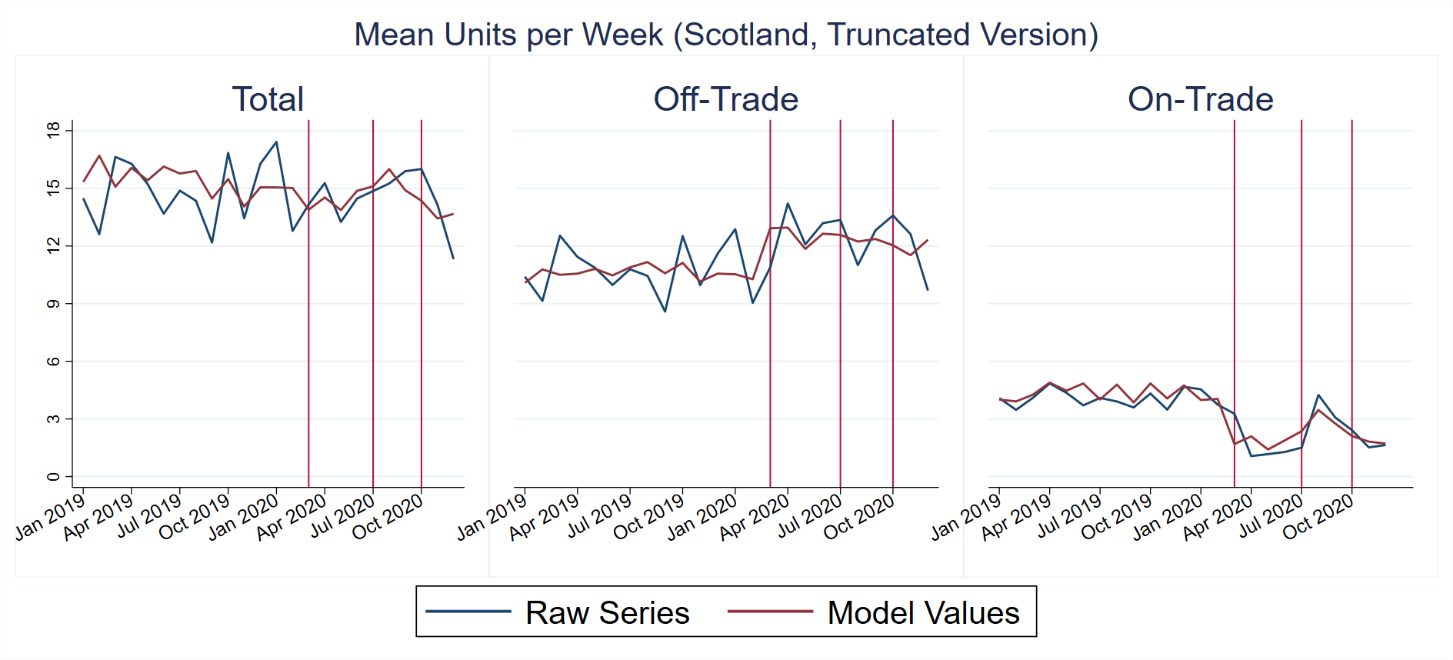


**Figure S20.** Mean Proportion Drinking >14 Units per week in Scotland (vertical lines from left to right = months where lockdown restrictions were introduced, eased and reintroduced)


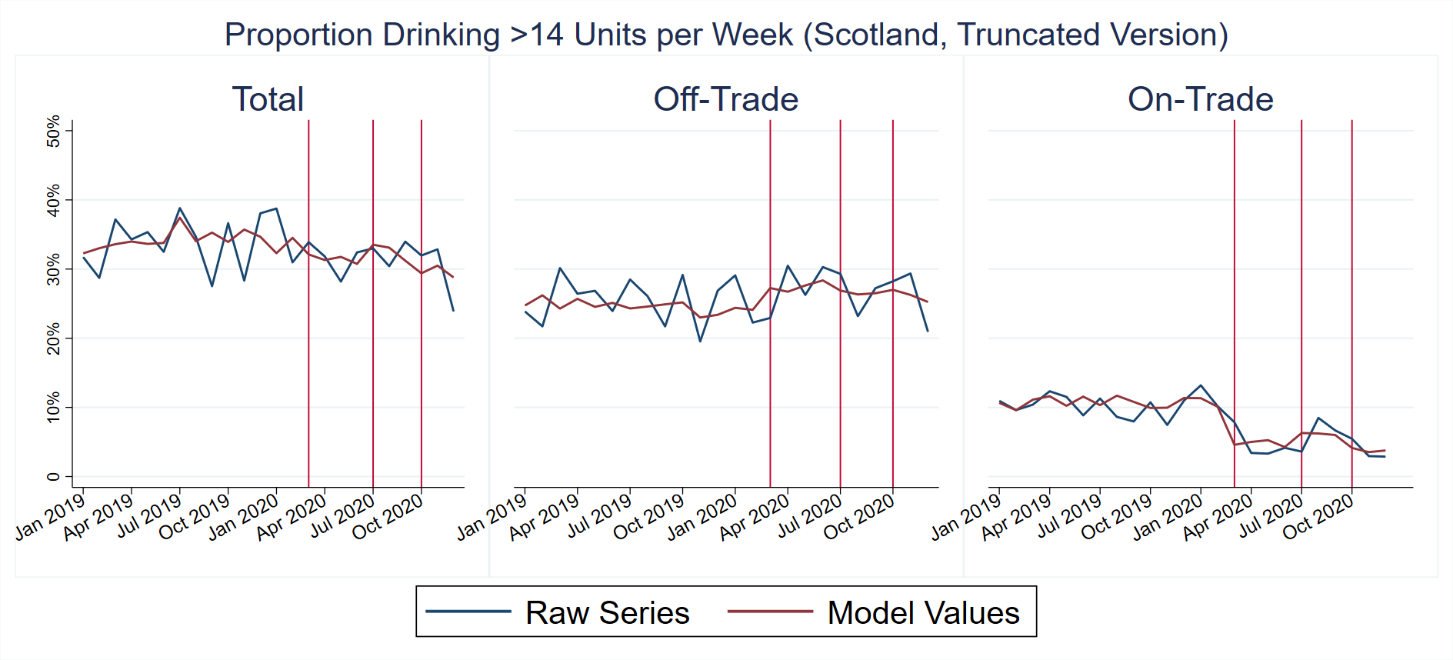


**Figure S21.** Mean heavy drinking occasions per week in Scotland (vertical lines from left to right = months where lockdown restrictions were introduced, eased and reintroduced)


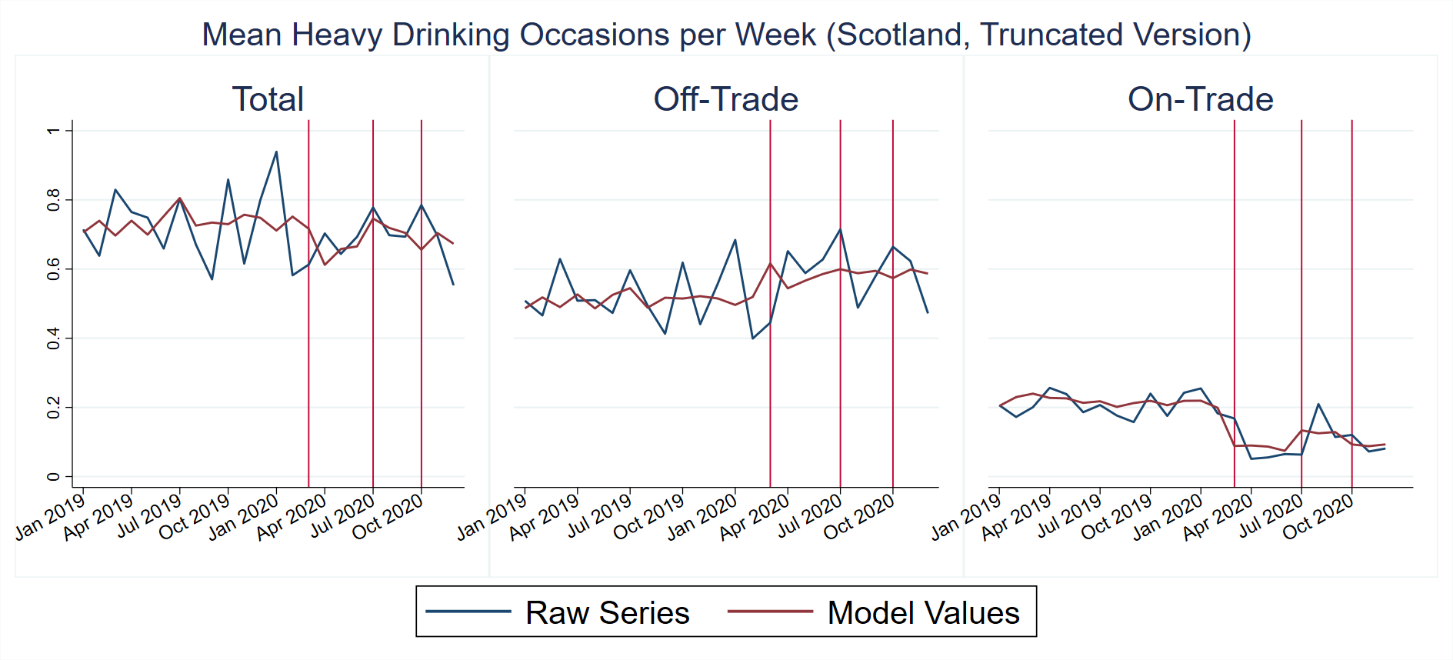


**Figure S22.** Mean drinking days per week in Scotland (vertical lines from left to right = months where lockdown restrictions were introduced, eased and reintroduced)


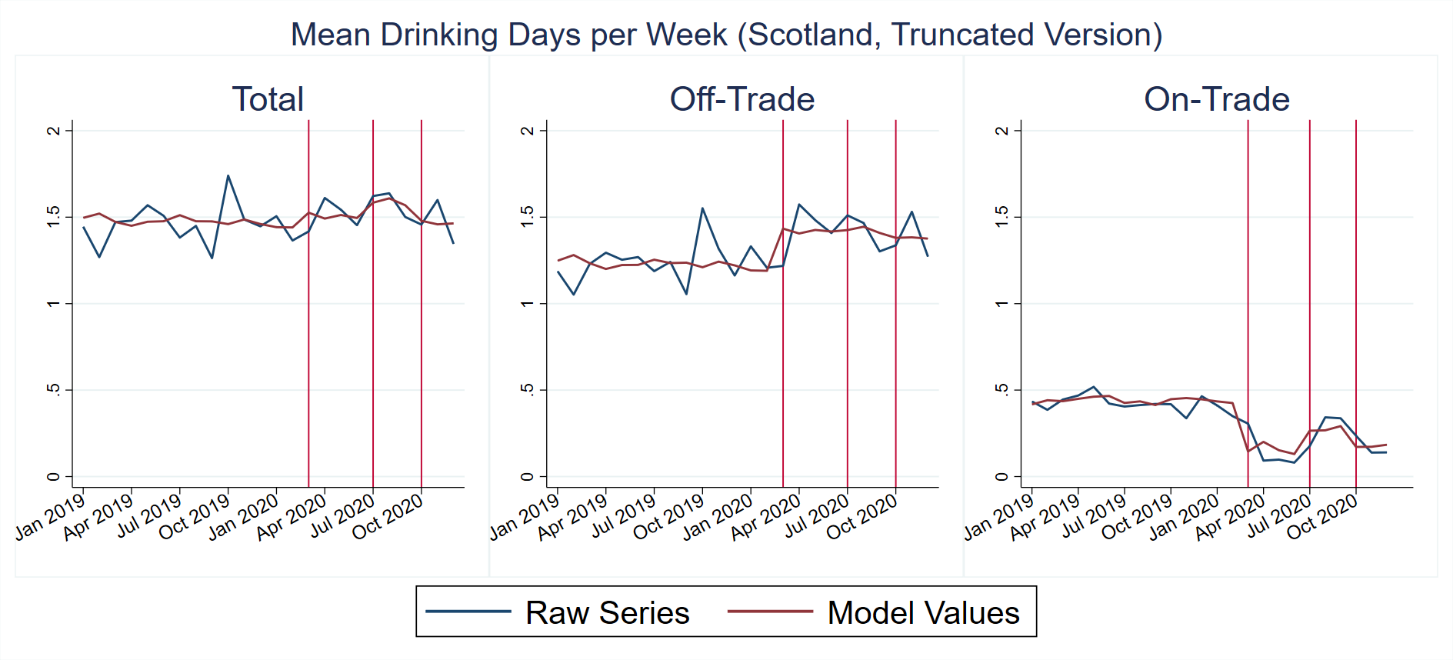


**Figure S23.** Mean drinking occasions per week by who with in Scotland (vertical lines from left to right = months where lockdown restrictions were introduced, eased and reintroduced)


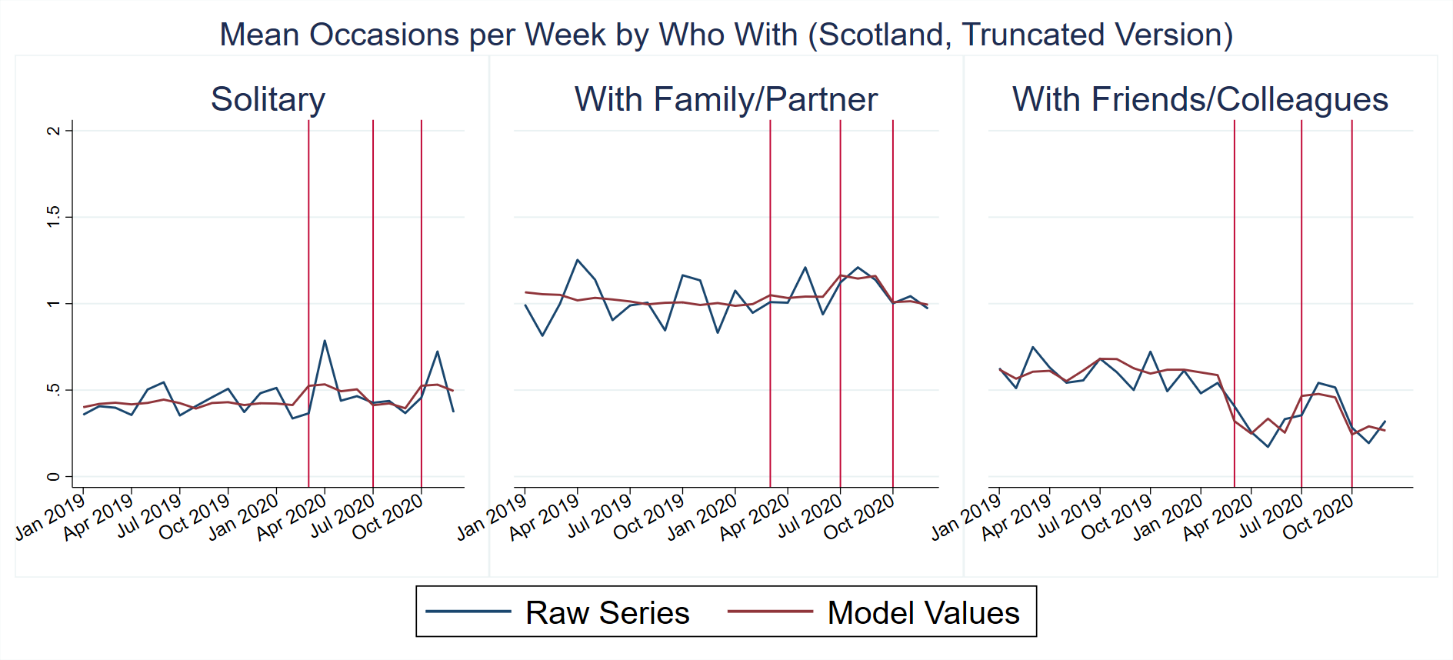


**Figure S24.** Mean drinking occasions per week by off-trade location in Scotland (vertical lines from left to right = months where lockdown restrictions were introduced, eased and reintroduced)


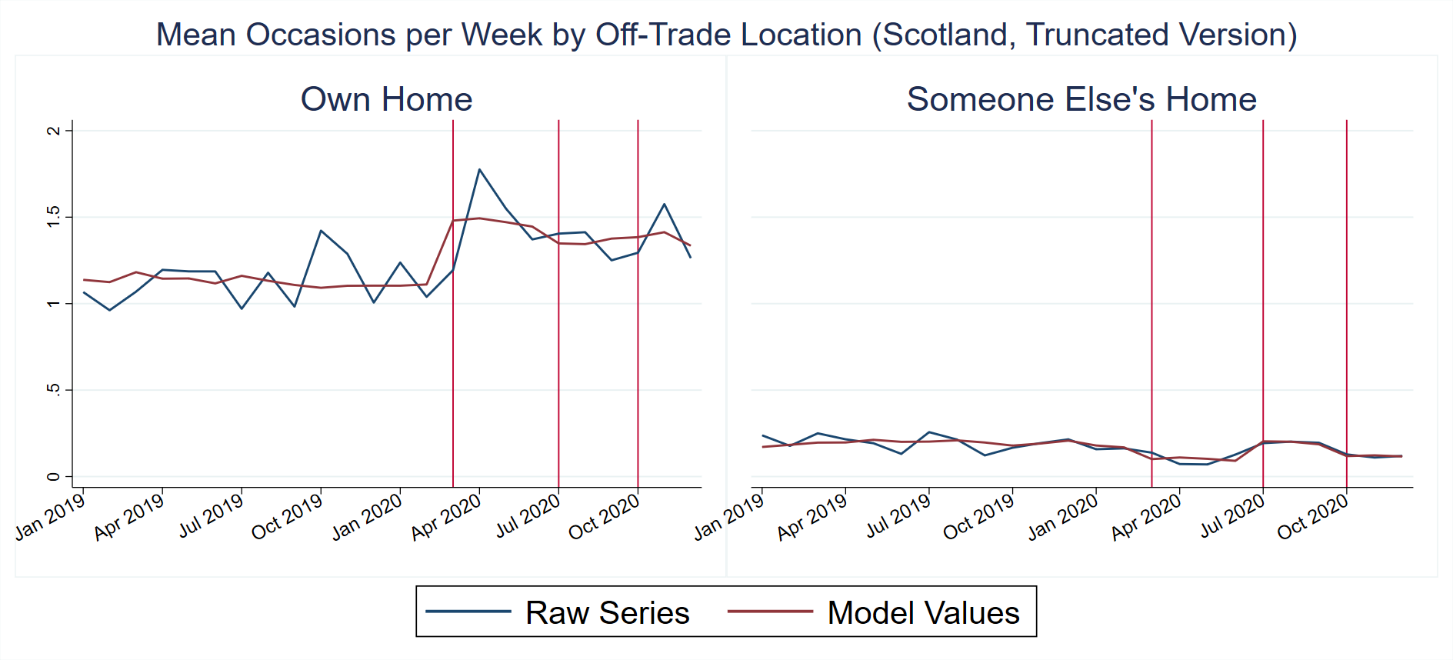


**Figure S25.** Mean start time of first drinking occasion per day in Scotland (vertical lines from left to right = months where lockdown restrictions were introduced, eased and reintroduced)


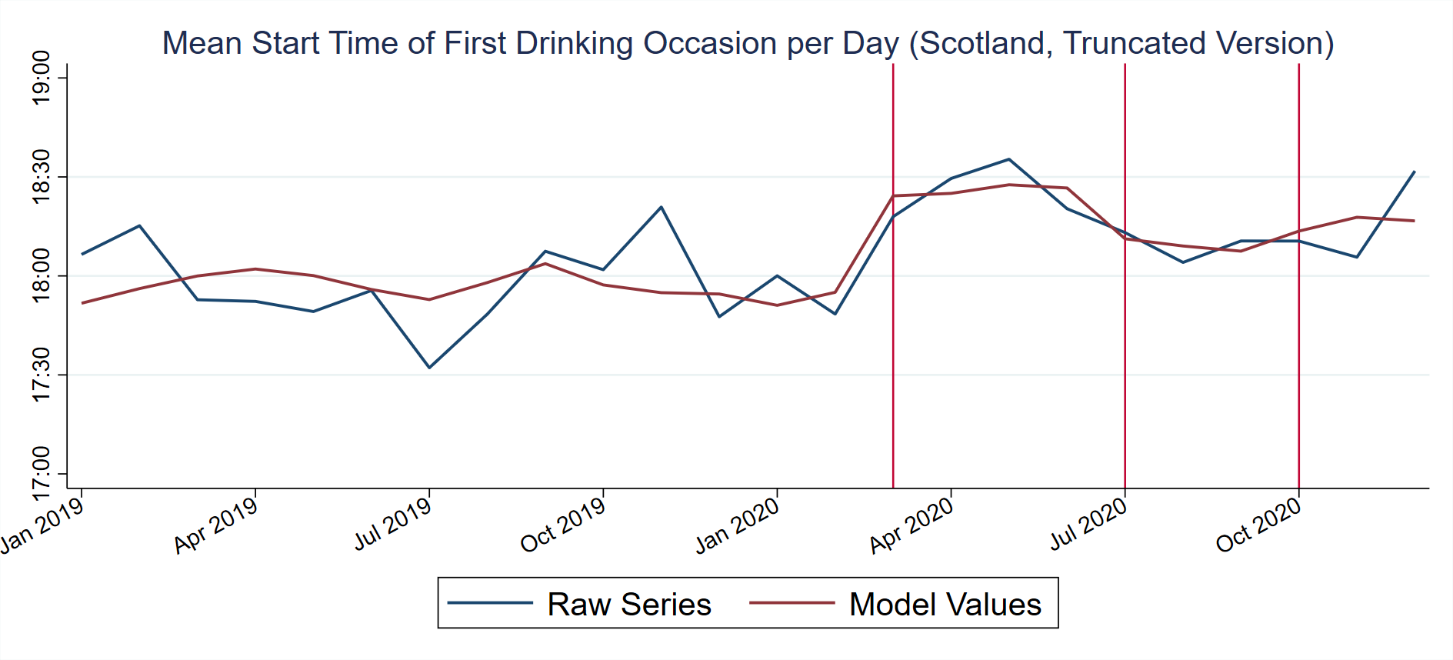


## RAW MONTHLY TIME-SERIES AND ITS MODEL VALUES (ENGLAND, TRUNCATED VERSIONS)

**Figure S26.** Mean units per week in England (vertical lines from left to right = months where lockdown restrictions were introduced, eased and reintroduced)


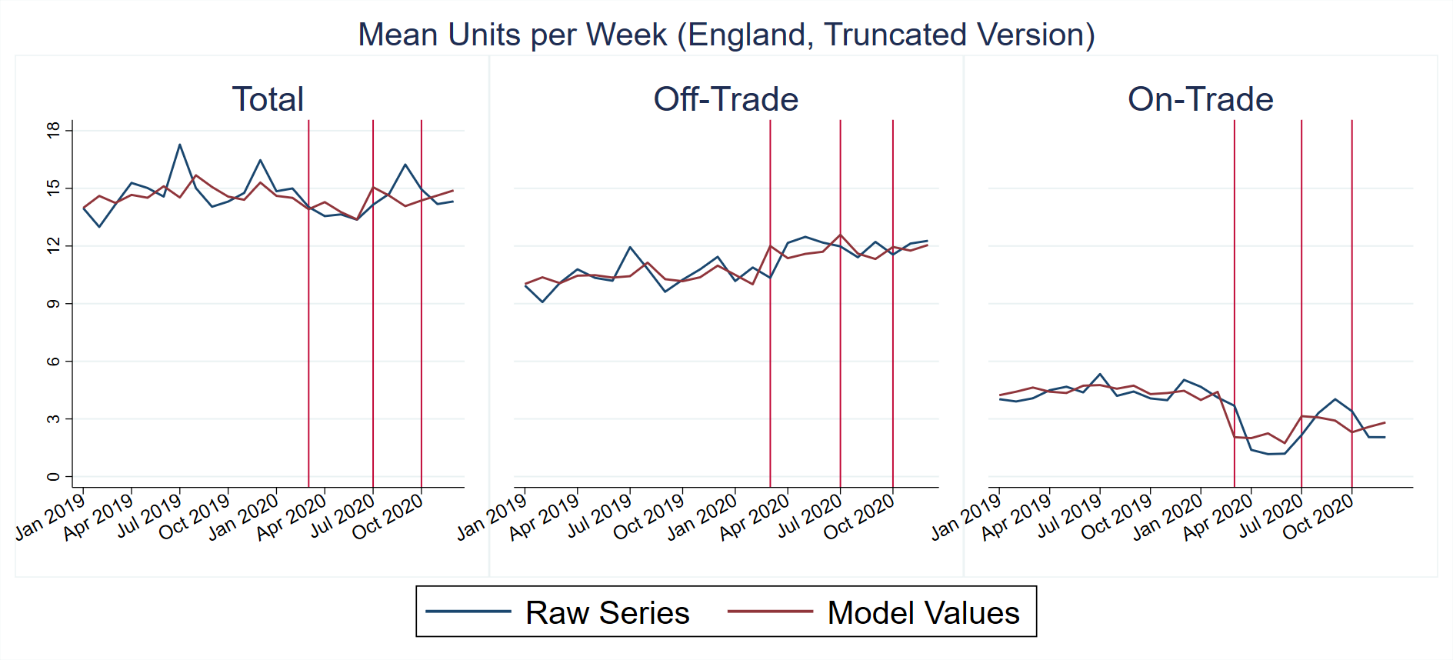


**Figure S27.** Mean Proportion Drinking >14 Units per week in England (vertical lines from left to right = months where lockdown restrictions were introduced, eased and reintroduced)


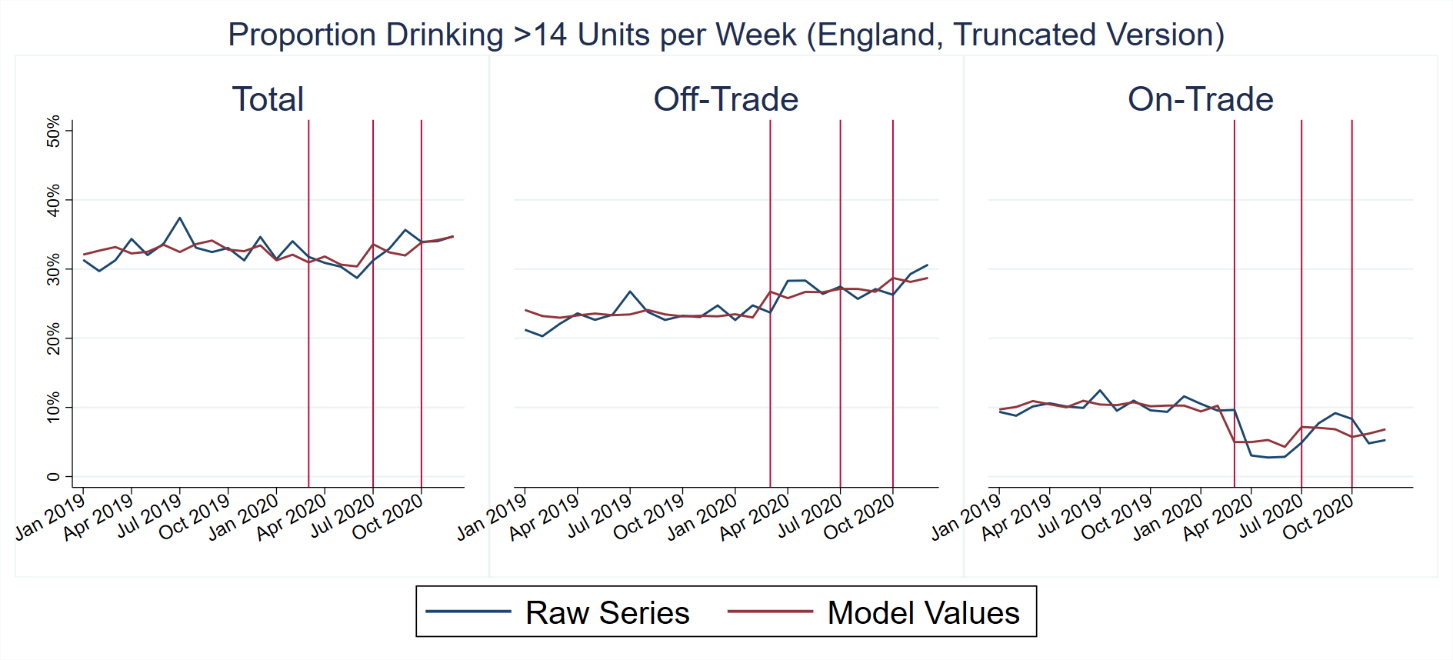


**Figure S28.** Mean heavy drinking occasions per week in England (vertical lines from left to right = months where lockdown restrictions were introduced, eased and reintroduced)


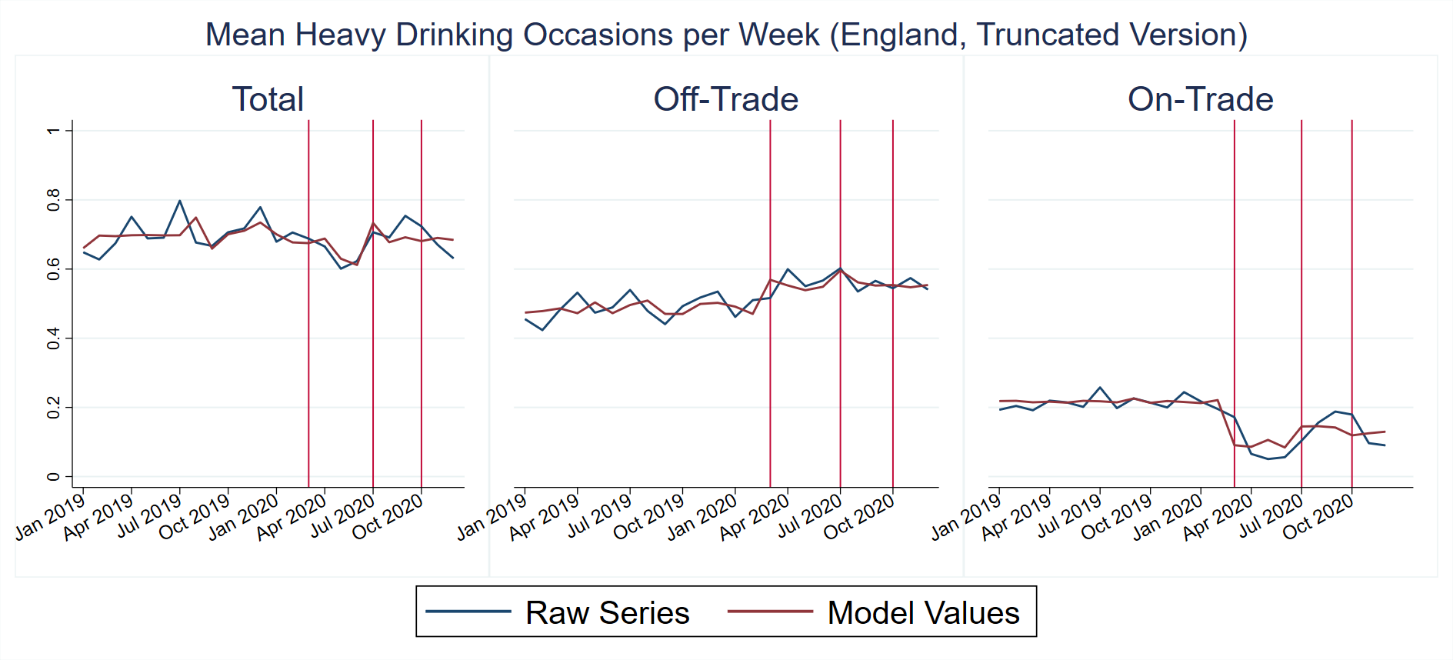


**Figure S29.** Mean drinking days per week in England (vertical lines from left to right = months where lockdown restrictions were introduced, eased and reintroduced)


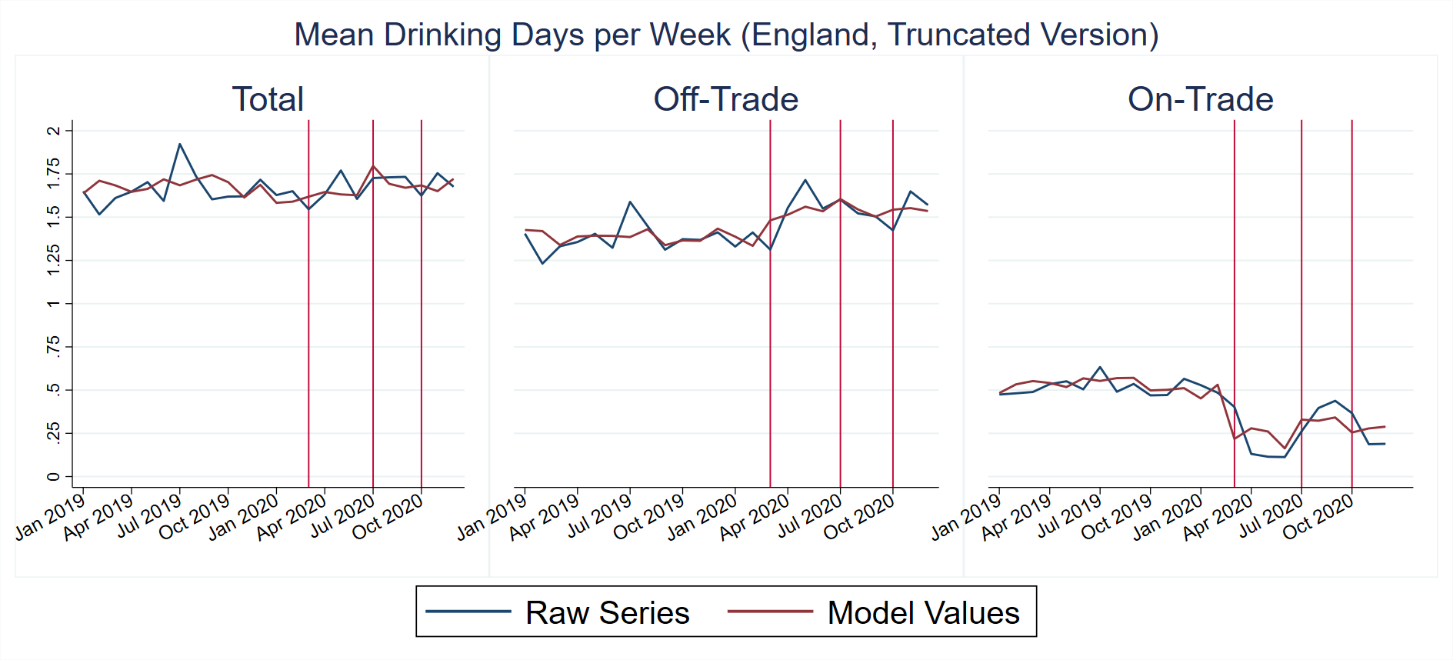


**Figure S30.** Mean drinking occasions per week by who with in England (vertical lines from left to right = months where lockdown restrictions were introduced, eased and reintroduced)


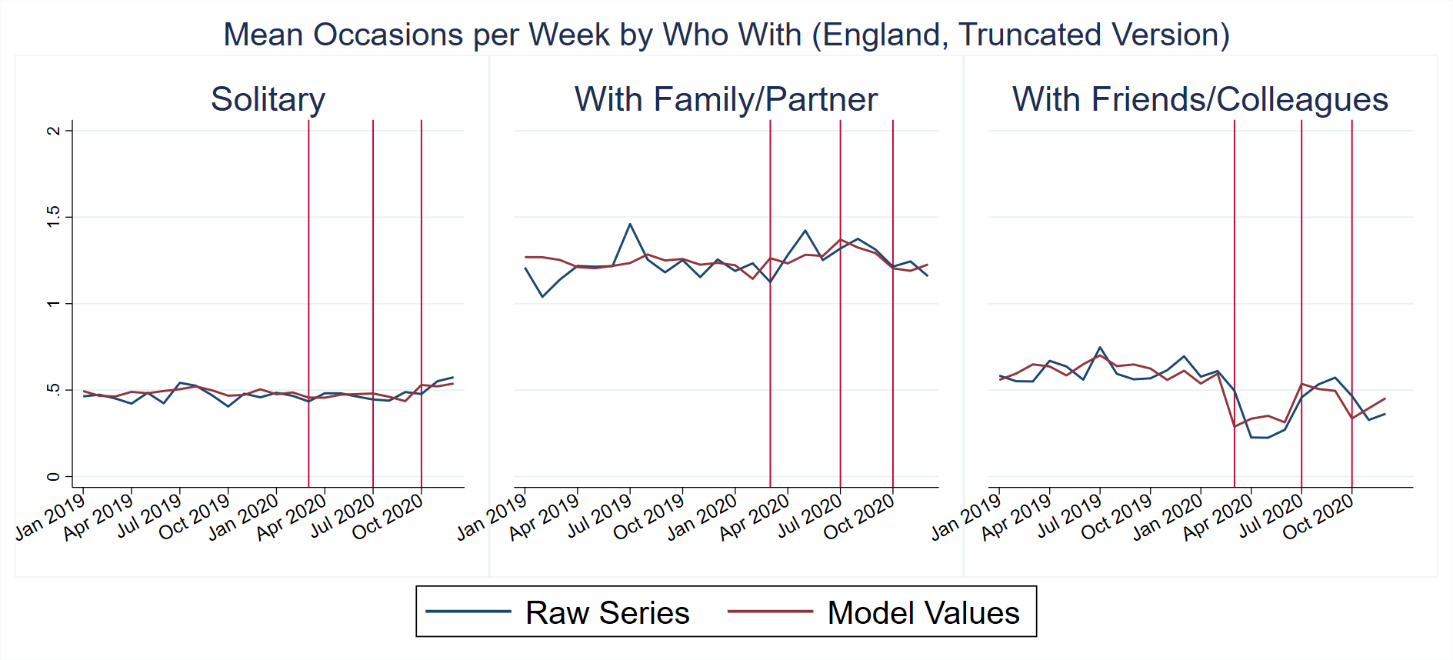


**Figure S31.** Mean drinking occasions per week by off-trade location in England (vertical lines from left to right = months where lockdown restrictions were introduced, eased and reintroduced)


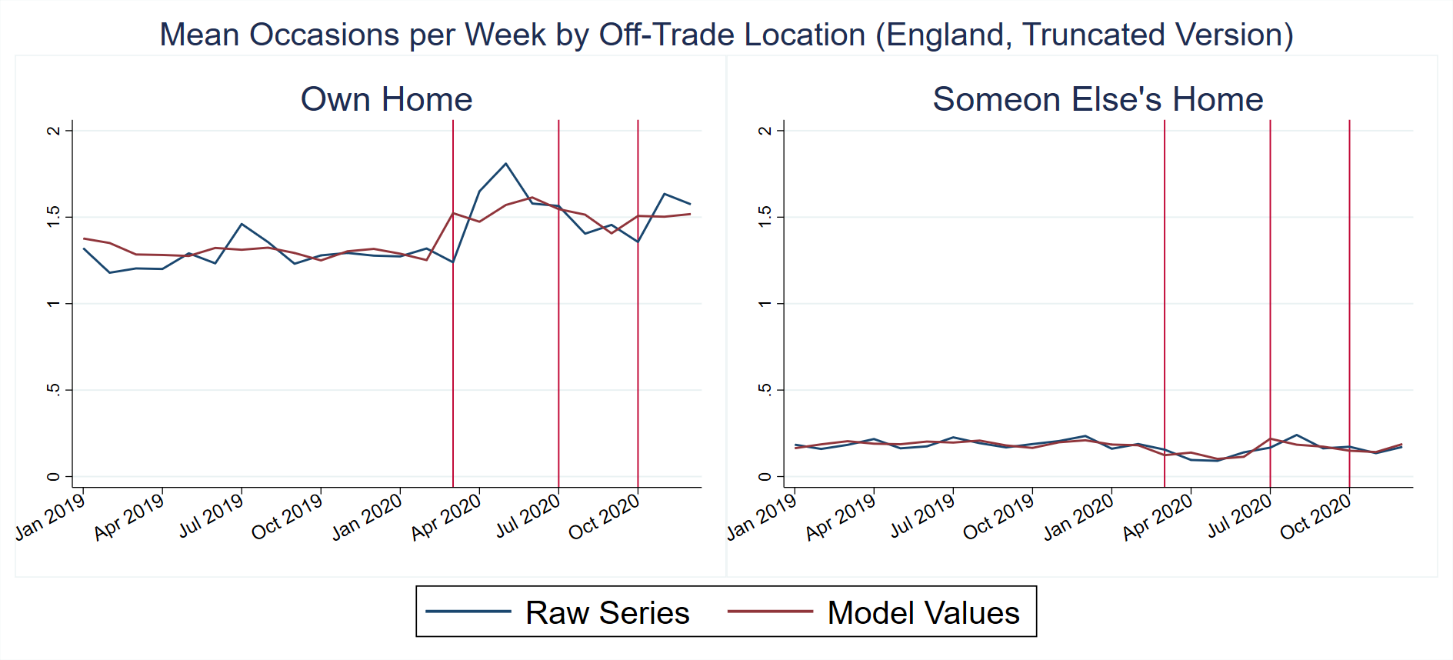


**Figure S32.** Mean start time of first drinking occasion per day in Scotland (vertical lines from left to right = months where lockdown restrictions were introduced, eased and reintroduced)


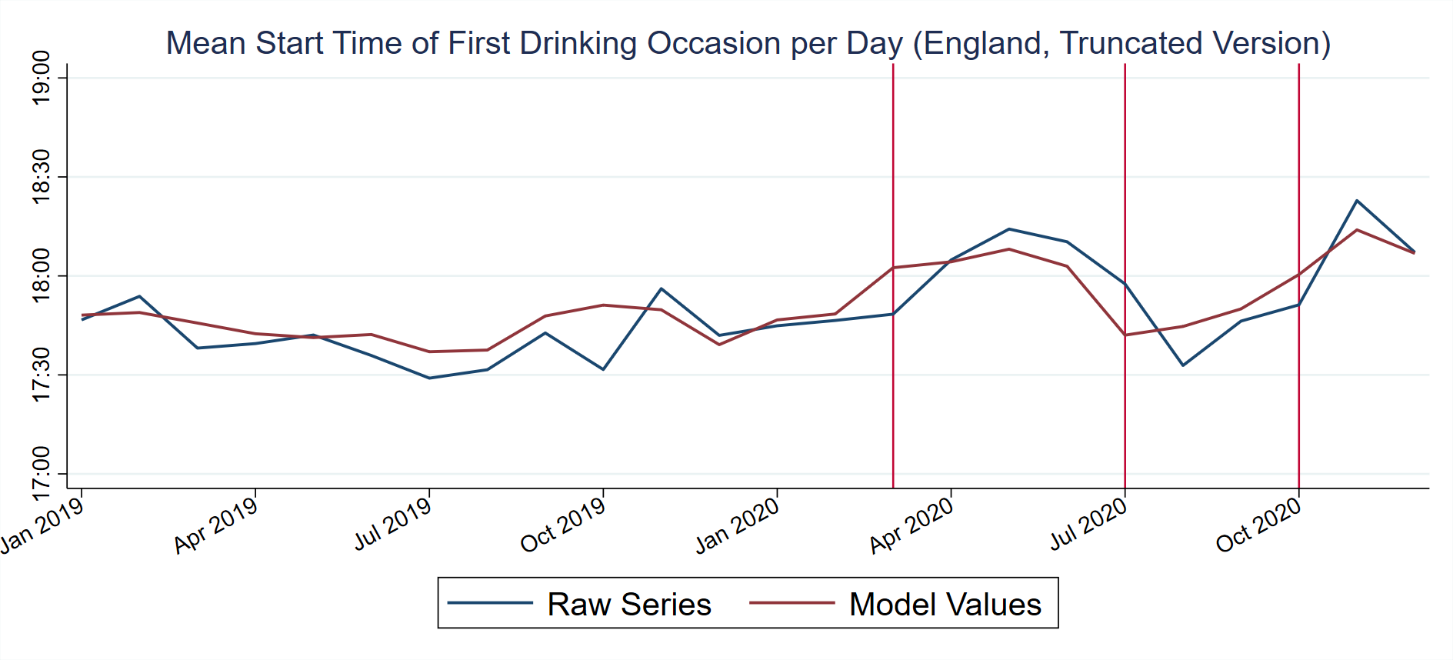

Supplement: Supplementary file 3 — Figure S7. Mean proportion drinking >14 units per week in Scotland (vertical lines from left to right = months where lockdown restrictions were introduced, eased and reintroduced) [file ADD-117-1622-s006.docx]
