# Supplementary material for: The impact of changes in COVID‐19 lockdown restrictions on alcohol consumption and drinking occasion characteristics in Scotland and England in 2020: an interrupted time‐series analysis
Source: Addiction. 2022 Feb 2;117(6):1622–39. doi: 10.1111/add.15794 (PMC9302640; doi:10.1111/add.15794)

# SUPPORTING INFORMATION APPENDIX D

## KERNEL DENSITY PLOTS CHECKING NORMALITY OF MODEL RESIDUALS (SCOTLAND)

**Figure S33.** Mean Units per Week (Table 3) kernel density plots showing model residuals with normal density overlaid


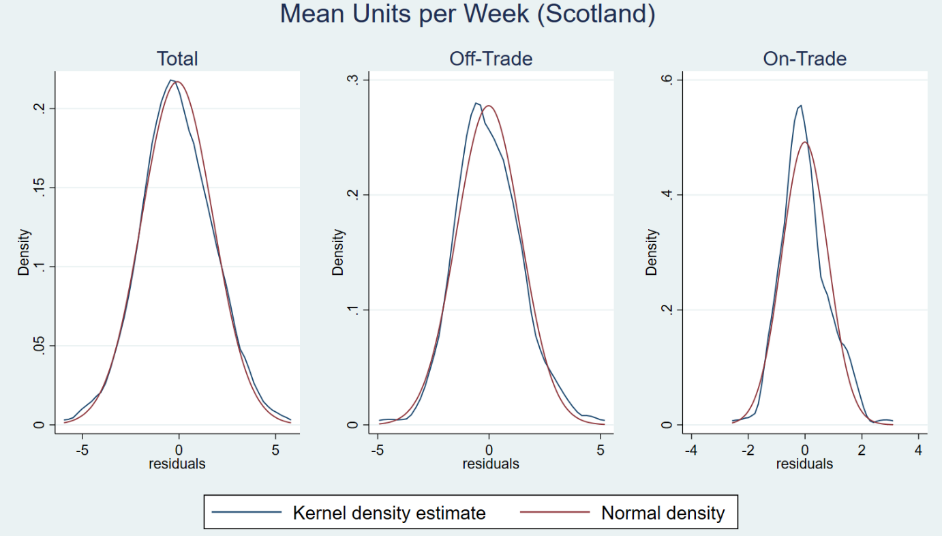


**Figure S34.** Proportion of individuals drinking >14 units per week (Table 3) kernel density plots showing model residuals with normal density overlaid


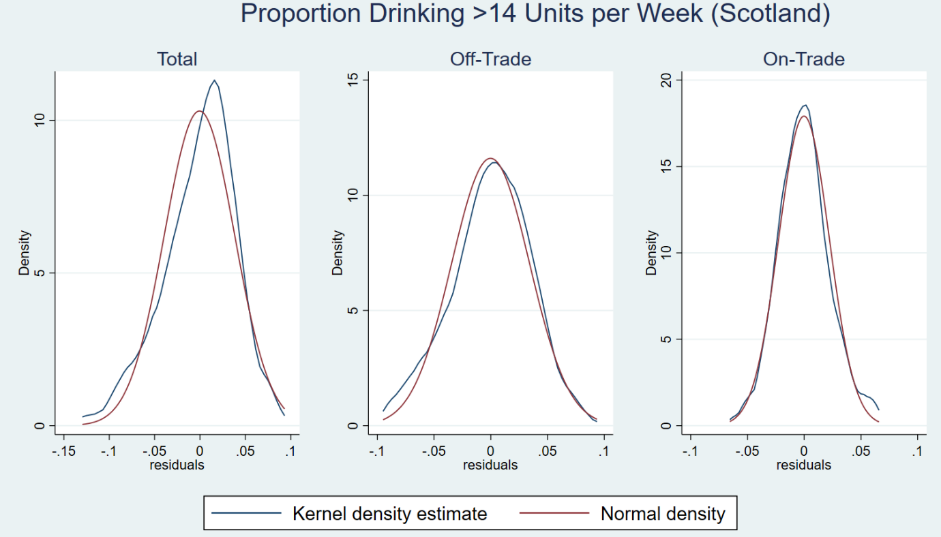


**Figure S35.** Mean number of heavy drinking occasions per week (Table 3) kernel density plots showing model residuals with normal density overlaid


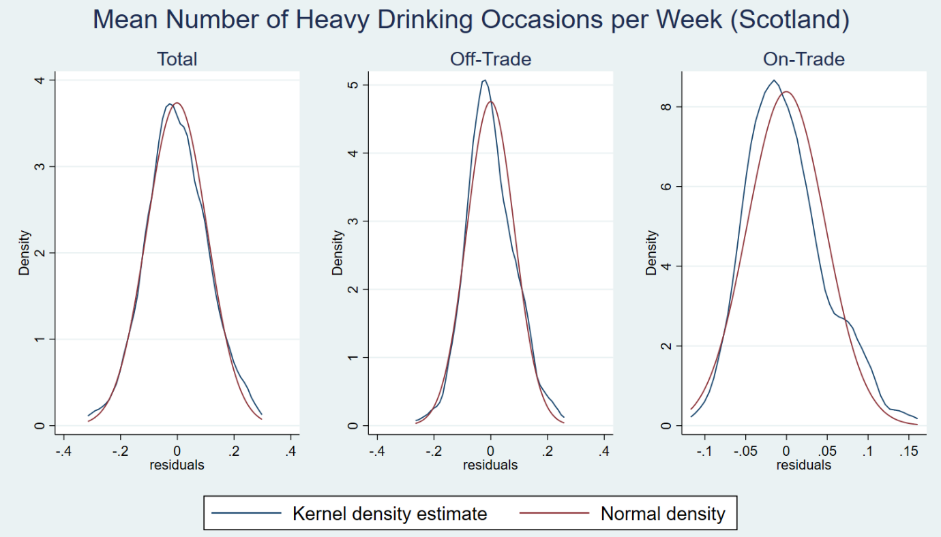


**Figure S36.** Mean number of drinking days per week (Table 3) kernel density plots showing model residuals with normal density overlaid


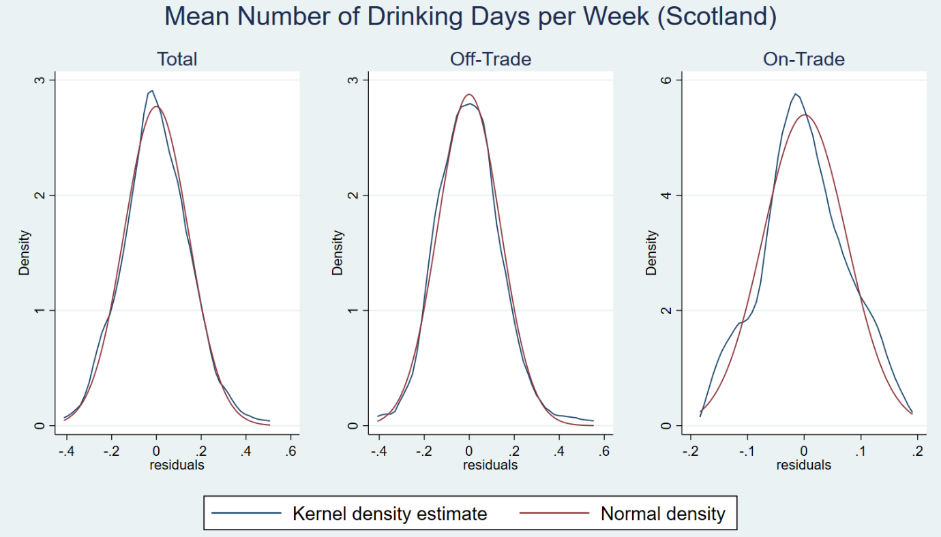


**Figure S37.** Mean number of solitary occasions per week (Table 5) kernel density plots showing model residuals with normal density overlaid


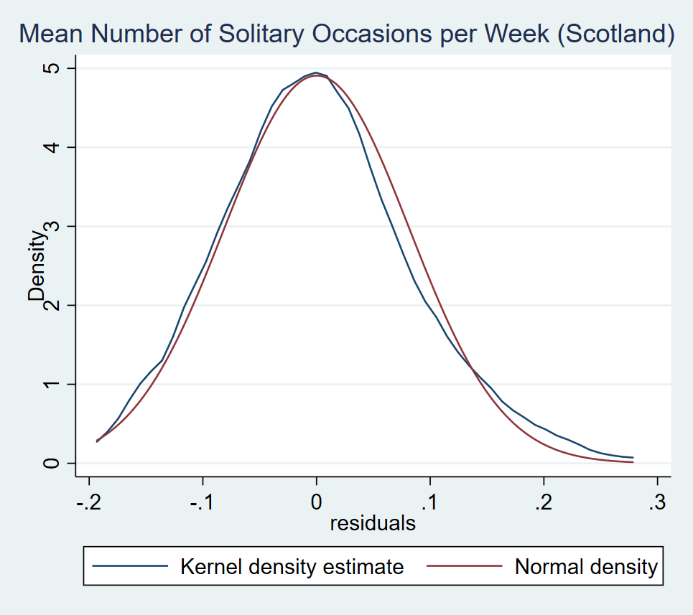


**Figure S38.** Mean number of occasions per week with family/partner (Table 5) kernel density plots showing model residuals with normal density overlaid


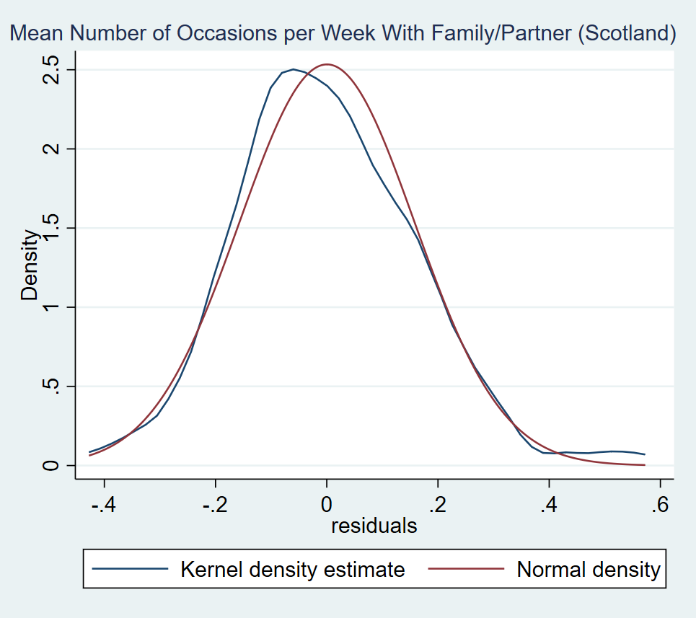


**Figure S39.** Mean number of occasions per week with friends/colleagues (Table 5) kernel density plots showing model residuals with normal density overlaid


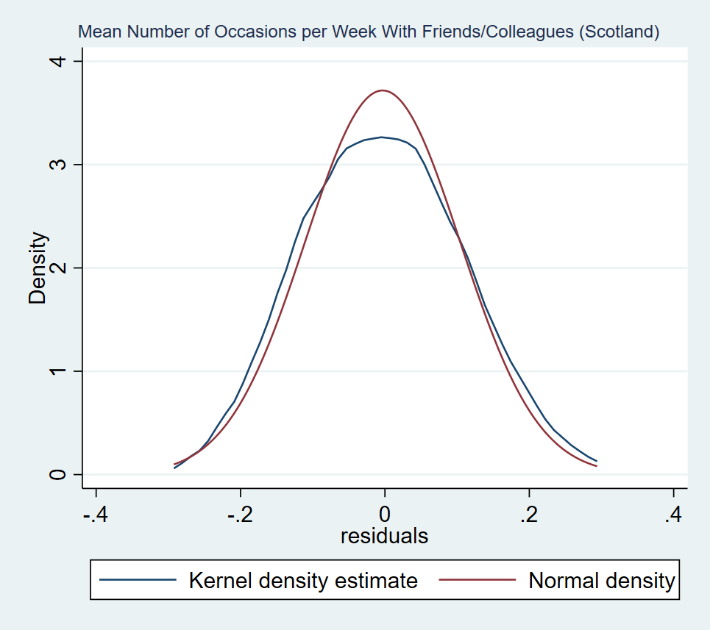


**Figure S40.** Mean number of occasions per week in own home (Table 5) kernel density plots showing model residuals with normal density overlaid


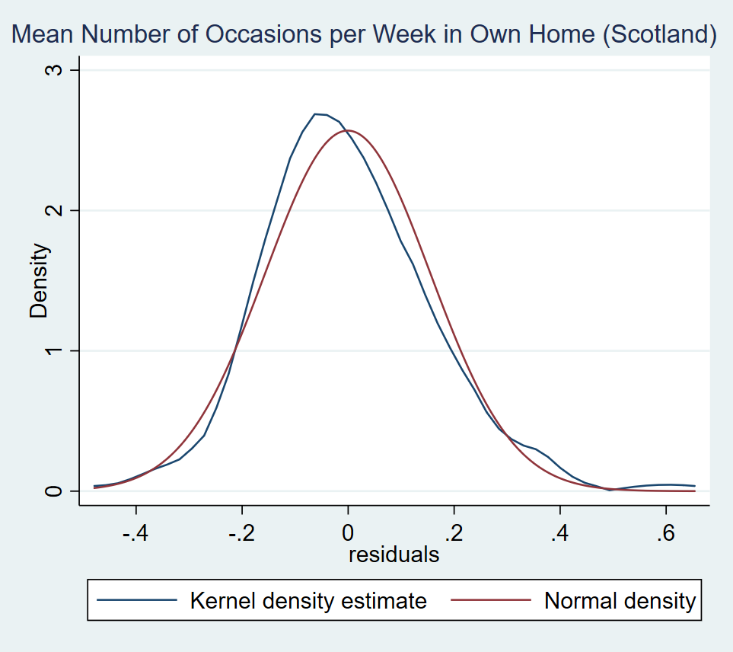


**Figure S41.** Mean number of occasions per week in someone else’s home (Table 5) kernel density plots showing model residuals with normal density overlaid


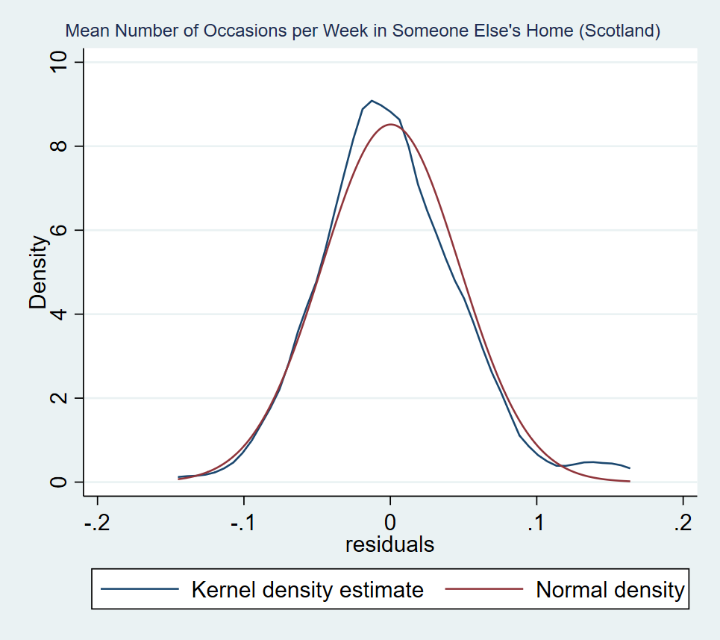


**Figure S42.** Mean start time of first drinking occasion (Table 5) kernel density plots showing model residuals with normal density overlaid


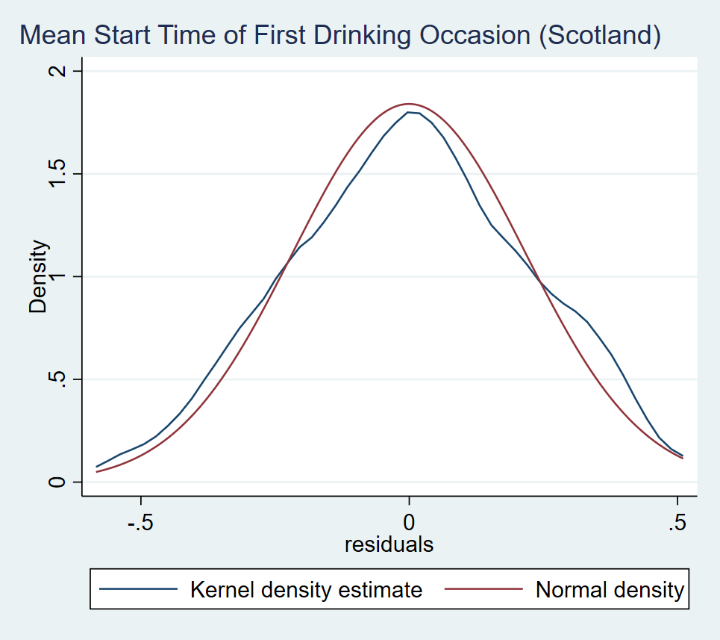


## KERNEL DENSITY PLOTS CHECKING NORMALITY OF MODEL RESIDUALS (ENGLAND)

**Figure S43.** Mean Units per Week (Table 4) kernel density plots showing model residuals with normal density overlaid


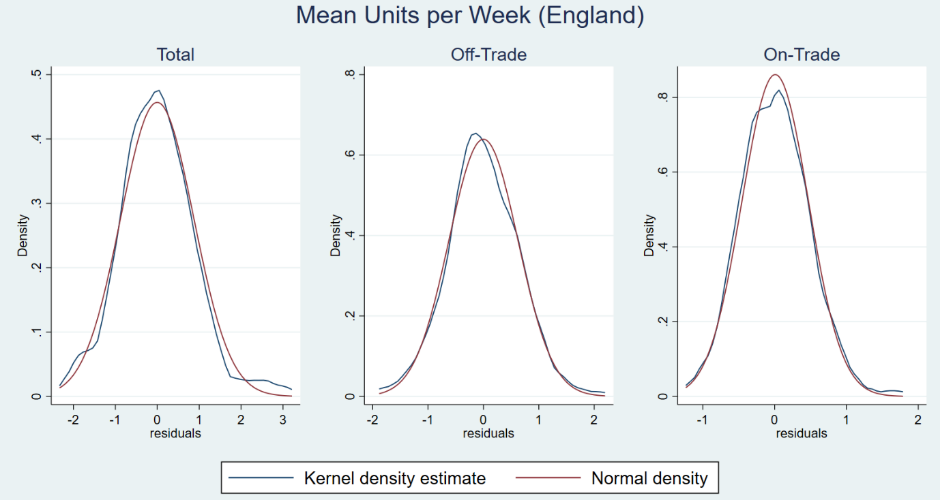


**Figure S44.** Proportion of individuals drinking >14 units per week (Table 4) kernel density plots showing model residuals with normal density overlaid


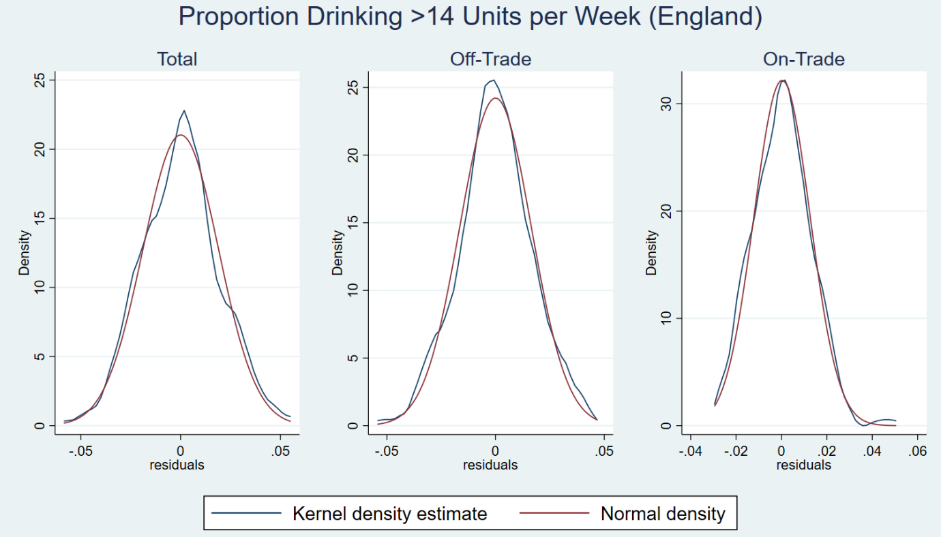


**Figure S45.** Mean number of heavy drinking occasions per week (Table 4) kernel density plots showing model residuals with normal density overlaid


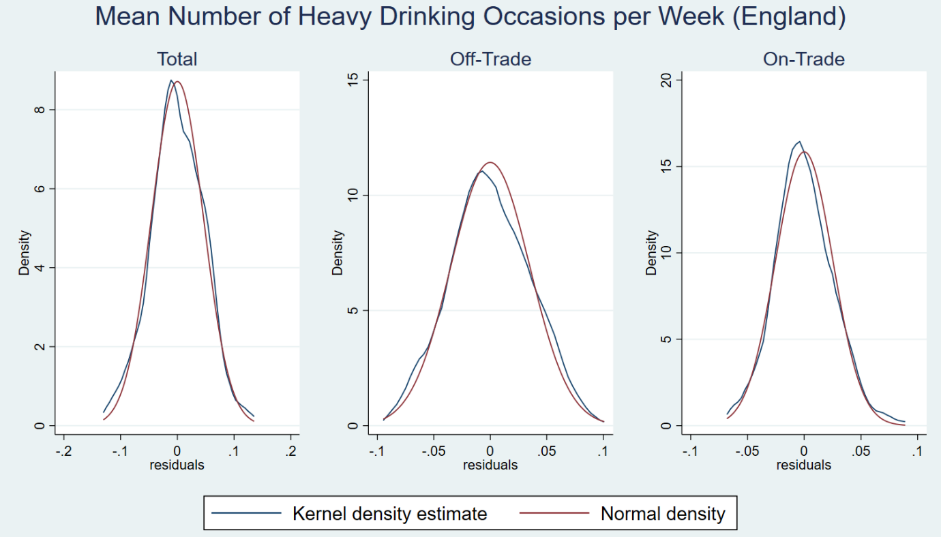


**Figure S46.** Mean number of drinking days per week (Table 4) kernel density plots showing model residuals with normal density overlaid


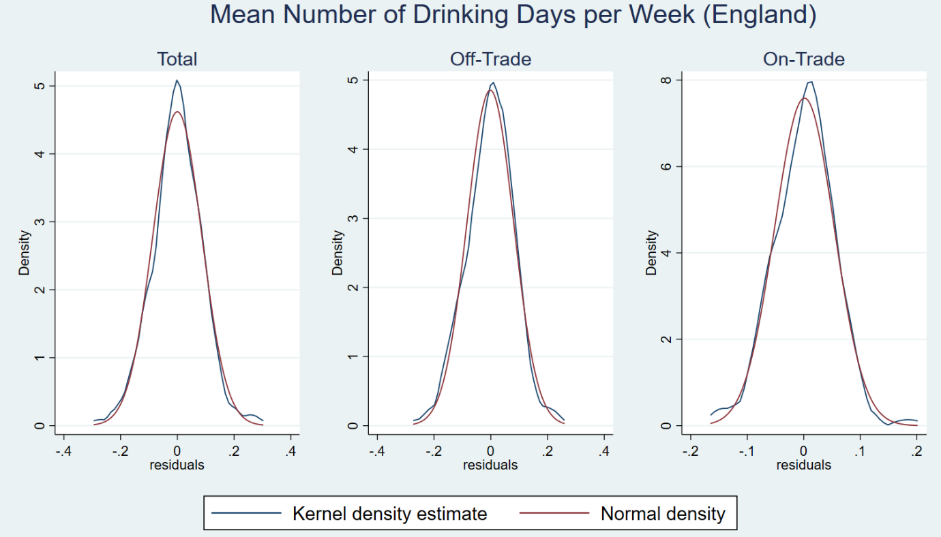


**Figure S47.** Mean number of solitary occasions per week (Table 6) kernel density plots showing model residuals with normal density overlaid


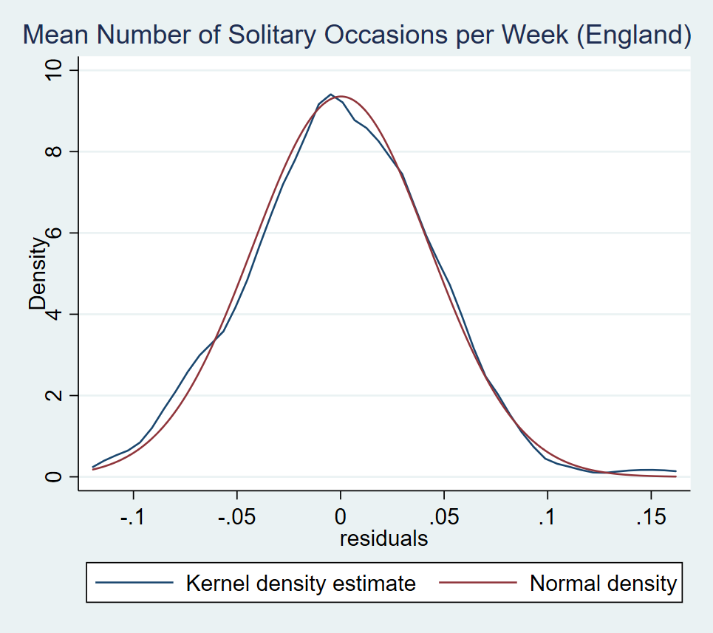


**Figure S48.** Mean number of occasions per week with family/partner (Table 6) kernel density plots showing model residuals with normal density overlaid


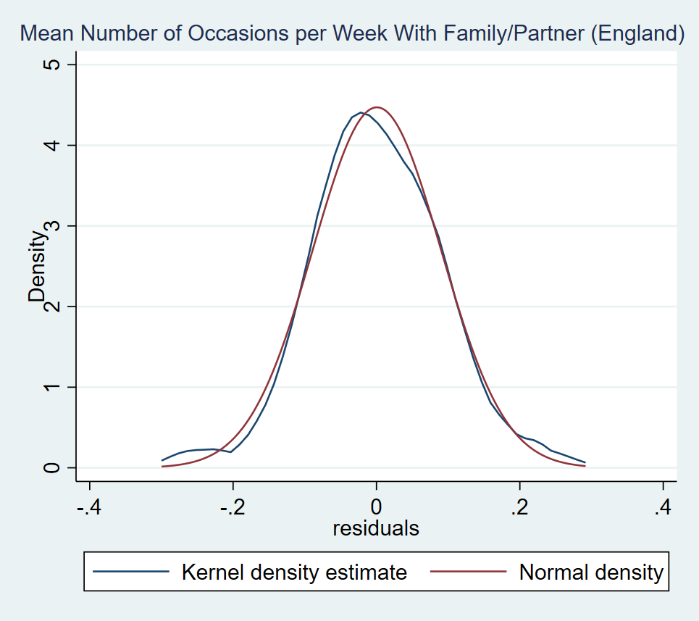


**Figure S49.** Mean number of occasions per week with friends/colleagues (Table 6) kernel density plots showing model residuals with normal density overlaid


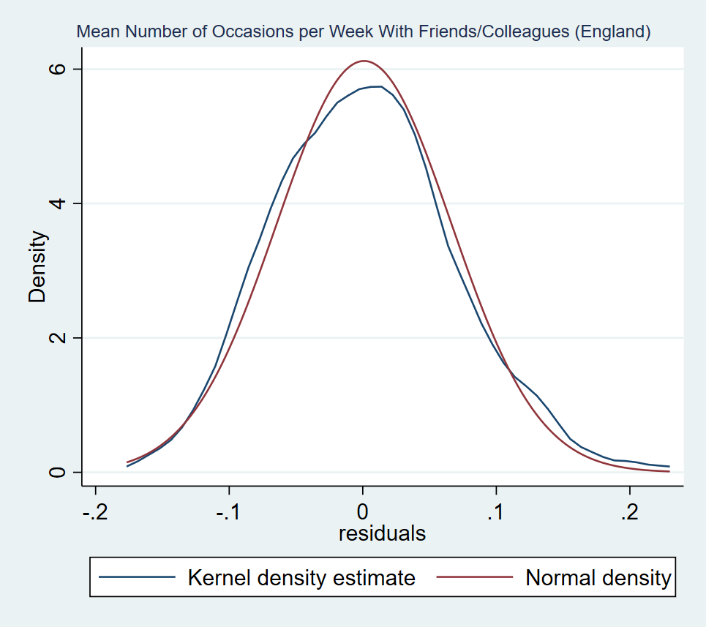


**Figure S50.** Mean number of occasions per week in own home (Table 6) kernel density plots showing model residuals with normal density overlaid


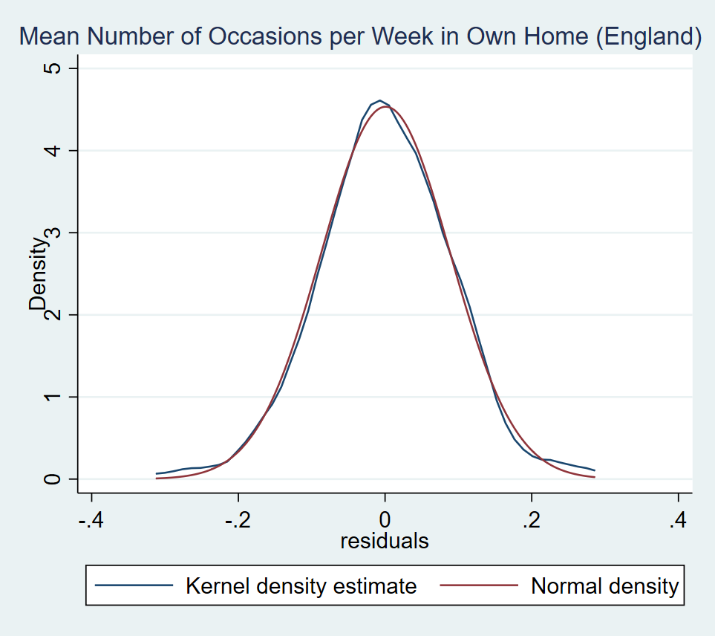


**Figure S51.** Mean number of occasions per week in someone else’s home (Table 6) kernel density plots showing model residuals with normal density overlaid


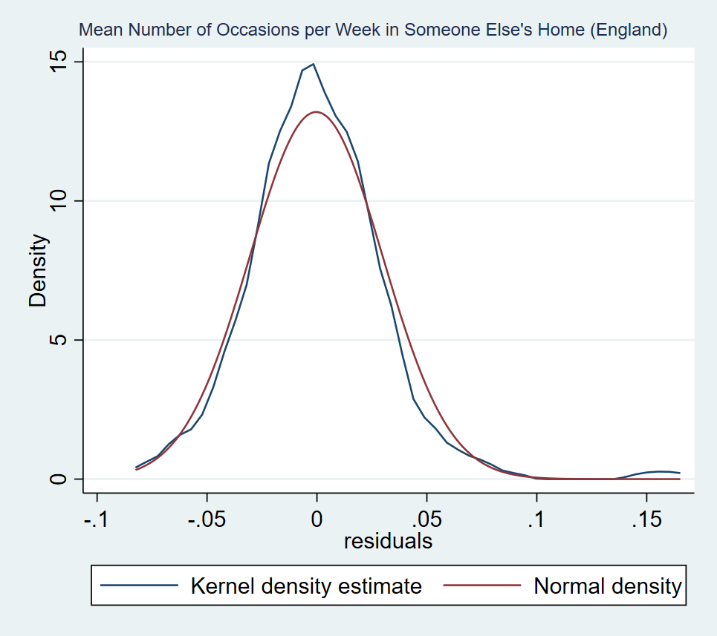


**Figure S52.** Mean start time of first drinking occasion (Table 6) kernel density plots showing model residuals with normal density overlaid


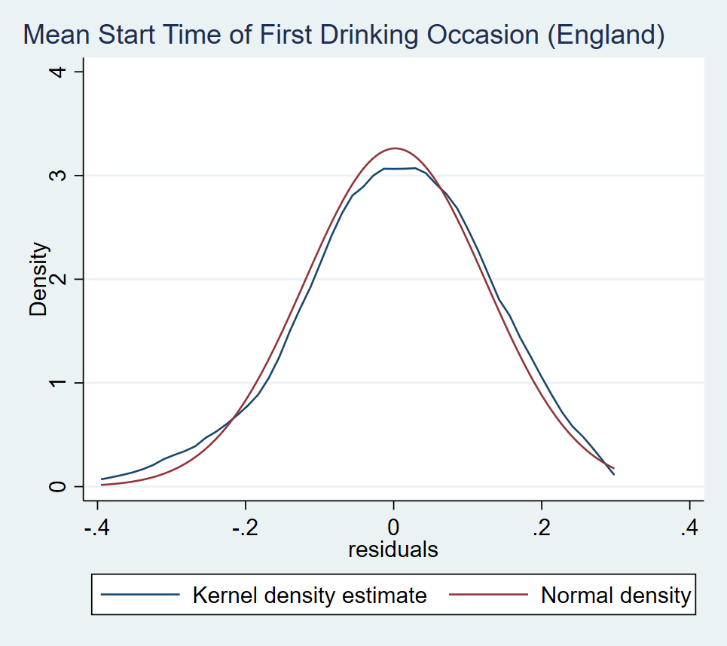

Supplement: Supplementary file 4 — Figure S33. Mean Units per Week (Table 3) kernel density plots showing model residuals with normal density overlaid [file ADD-117-1622-s002.docx]
