# Supplementary material for: The impact of changes in COVID‐19 lockdown restrictions on alcohol consumption and drinking occasion characteristics in Scotland and England in 2020: an interrupted time‐series analysis
Source: Addiction. 2022 Feb 2;117(6):1622–39. doi: 10.1111/add.15794 (PMC9302640; doi:10.1111/add.15794)
Supplement: Supplementary file 5 — Table S1. Mean units per week (Table 3) portmanteau test checking residuals resemble white noise [file ADD-117-1622-s004.docx]

# SUPPORTING INFORMATION APPENDIX E

## PORTMANTEAU TESTS TO CHECK THAT MODEL RESIDUALS RESEMBLE WHITE NOISE (SCOTLAND)

**Table S1.** Mean units per week (Table 3) portmanteau test checking residuals resemble white noise

**Table S2.** Proportion of individuals drinking >14 units per week (Table 3) portmanteau test checking residuals resemble white noise

**Table S3.** Mean number of heavy drinking occasions per week (Table 3) portmanteau test checking residuals resemble white noise

**Table S4.** Mean number of drinking days per week (Table 3) portmanteau test checking residuals resemble white noise

**Table S5.** Mean number of solitary occasions per week (Table 5) portmanteau test checking residuals resemble white noise

**Table S6.** Mean number of occasions per week with family/partner (Table 5) portmanteau test checking residuals resemble white noise

**Table S7.** Mean number of occasions per week with friends/colleagues (Table 5) portmanteau test checking residuals resemble white noise

**Table S8.** Mean number of occasions per week in own home (Table 5) portmanteau test checking residuals resemble white noise

**Table S9.** Mean number of occasions per week in someone else’s home (Table 5) portmanteau test checking residuals resemble white noise

**Table S10.** Mean start time of first drinking occasion (Table 5) portmanteau test checking residuals resemble white noise

## KERNEL DENSITY PLOTS CHECKING NORMALITY OF MODEL RESIDUALS (ENGLAND)

**Table S11.** Mean units per week (Table 4) portmanteau test checking residuals resemble white noise

**Table S12.** Proportion of individuals drinking >14 units per week (Table 4) portmanteau test checking residuals resemble white noise

**Table S13.** Mean number of heavy drinking occasions per week (Table 4) portmanteau test checking residuals resemble white noise

**Table S14.** Mean number of drinking days per week (Table 4) portmanteau test checking residuals resemble white noise

**Table S15.** Mean number of solitary occasions per week (Table 6) portmanteau test checking residuals resemble white noise

**Table S16.** Mean number of occasions per week with family/partner (Table 6) portmanteau test checking residuals resemble white noise

**Table S17.** Mean number of occasions per week with friends/colleagues (Table 6) portmanteau test checking residuals resemble white noise

**Table S18.** Mean number of occasions per week in own home (Table 6) portmanteau test checking residuals resemble white noise

**Table S19.** Mean number of occasions per week in someone else’s home (Table 6) portmanteau test checking residuals resemble white noise

**Table S20.** Mean start time of first drinking occasion (Table 6) portmanteau test checking residuals resemble white noise
