# Supplementary material for: The impact of changes in COVID‐19 lockdown restrictions on alcohol consumption and drinking occasion characteristics in Scotland and England in 2020: an interrupted time‐series analysis
Source: Addiction. 2022 Feb 2;117(6):1622–39. doi: 10.1111/add.15794 (PMC9302640; doi:10.1111/add.15794)
Supplement: Supplementary file 6 — Data S3. Supporting Information [file ADD-117-1622-s001.docx]

# SUPPORTING INFORMATION APPENDIX F

**SENSITIVITY ANALYSIS CONTROLLING FOR INTRODUCTION OF MINIMUM UNIT PRICING IN SCOTLAND MODELS ON ALCOHOL CONSUMPTION**

Estimated impact of the introducing, easing and reintroducing COVID-19 lockdown restrictions on alcohol consumption measures in Scotland (step changes), controlling for May 2018 introduction of Minimum Unit Pricing (MUP)

|  | *(1)*  *Mean Units per Week* | | *(2)*  *Proportion of Individuals Drinking >14 Units per Week* | | *(3)*  *Mean Number of Heavy Drinking Occasions per Week* | | *(4)*  *Mean Number of Drinking Days per week* | |
| --- | --- | --- | --- | --- | --- | --- | --- | --- |
| **Total** | **B [95% CI]** | **P** | **B [95% CI]** | **P** | **B [95% CI]** | **P** | **B [95% CI]** | **P** |
| *Initial Lockdown*  *Restrictions Eased*  *Some Restrictions Reintroduced*  *MUP*  *AR Terms* | -0.10  [-4.90, 4.69]  1.36  [-6.39, 9.11]  -1.72  [-8.27, 4.82]  -1.12  [-2.34, 0.08]  **L25**: -0.25  [-0.04, -0.02] | 0.966  0.730  0.606  0.069  0.008 | -0.01  [-0.09, 0.08]  0.004  [-0.11, 0.12]  -0.03  [-0.12, 0.06]  -0.04  [-0.06, -0.01]  **L30**: -0.22  [-0.43, -0.01] | 0.884  0.938  0.523  0.003  0.040 | -0.02  [-0.17, 0.13]  0.05  [-0.53, 0.63]  -0.02  [-0.61, 0.56]  -0.08  [-0.14, -0.02]  **L20**: -0.21  [-0.40, -0.03]  **L30**: -0.25  [-0.43, -0.06] | 0.780  0.868  0.938  0.007  0.025  0.011 | 0.12  [-0.11, 0.36]  0.10  [-0.43, 0.63]  -0.08  [-0.43, 0.63]  -0.11  [-0.21, -0.02]  **L18**: -0.23  [-0.40, -0.06] | 0.297  0.722  0.773  0.022  0.007 |
| **Off-Trade** |  |  |  |  |  |  |  |  |
| *Initial Lockdown*  *Restrictions Eased*  *Some Restrictions Reintroduced*  *MUP*  *AR Terms* | 2.51  [0.60, 4.42]  -0.16  [-3.03, 2.70]  -0.36  [-3.17, 2.45]  -1.17  [-1.17, 0.62]  **L14**: -0.15  [-0.37, 0.07] | 0.010  0.910  0.801  0.551  0.183 | 0.04  [0.003, 0.07]  -0.01  [-0.08, 0.06]  -0.01  [-0.08, 0.07]  -0.01  [-0.02, 0.01]  **L25**: -0.19  [-0.40, 0.02]  **L39**: 0.15  [-0.08, 0.39] | 0.029  0.836  0.883  0.477  0.082  0.200 | 0.08  [-0.01, 0.18]  0.02  [-0.11, 0.15]  -0.01  [-0.14, 0.12]  -0.02  [-0.07, 0.03]  **L25**: -0.20  [-0.39, -0.01] | 0.088  0.786  0.838  0.414  0.044 | 0.25  [0.10, 0.40]  0.02  [-0.28, 0.31]  -0.11  [-0.34, 0.32]  -0.04  [-0.14, 0.05  **L18**: -0.20  [-0.37, -0.02] | 0.001  0.919  0.949  0.326  0.028 |
| **On-Trade** |  |  |  |  |  |  |  |  |
| *Initial Lockdown*  *Restrictions Eased*  *Some Restrictions Reintroduced*  *MUP*  *AR Terms* | -2.29  [-3.33, -1.25]  1.43  [0.31, 2.56]  -1.06  [-3.82, 1.70]  -1.11  [-1.69, -0.52]  **L20**: -0.23  [-0.39, -0.08]  **L23**: -0.25  [-0.43, -0.06] | <0.001  0.012  0.452  <0.001  0.003  0.010 | -0.05  [-0.08, -0.02]  0.02  [-0.02, 0.06]  -0.02  [-0.07, 0.03]  -0.02  [-0.04, -0.01]  **L23**: -0.28  [-0.46, -0.10] | 0.001  0.392  0.461  0.007  0.002 | -0.11  [-0.17, -0.06]  -0.05  [-0.01, 0.11]  -0.04  [-0.19, 0.12]  -0.06  [-0.09, -0.02]  **L20**: -0.24  [-0.41, -0.08] | <0.001  0.103  0.650  0.001  0.004 | -0.22  [-0.30, -0.14]  0.14  [0.04, 0.24]  -0.13  [-0.19, 0.06]  -0.13  [-0.19, -0.07]  **L1**: 0.17  [-0.001, 0.34]  -**L20**: 0.21  [-0.39, -0.02]  **L38**: 0.21  [0.03, 0.39] | <0.001  0.07  0.187  <0.001  0.052  0.027  0.022 |

*Notes*: estimates are adjusted for autocorrelation, seasonality and trend. B=coefficient, 95% CI=95% confidence intervals and P=p-value. ‘*Initial Lockdown*’ includes all diary weeks from March 2020, ‘*Restrictions Eased*’ includes all diary weeks from July 2020 (when on-trade premises reopened and restrictions on visiting other households were relaxed), and ‘*Some Restrictions Reintroduced*’ includes all diary weeks from October 2020 (when local restrictions and the tier system were introduced. Results for the AR terms and seasonal AR terms included in each model are reported underneath the main results. L refers to number of lags, and these were selected following an iterative process involving ACF/PACF plots and model fit statistics.
